# Optimization of phenolic extraction method and *in vitro* bioaccessibility of microencapsulated pigmented rice bran extracts and their antioxidant and anticancer properties

**DOI:** 10.1016/j.fhfh.2025.100221

**Published:** 2025-06

**Authors:** Rhowell Navarro Tiozon, Glenn Vincent P. Ong, Kristel June D. Sartagoda, Sheba Mae M. Duque, Saleh Alseekh, Aldrin P. Bonto, Shem Gempesaw, Vipin Pratap, Florencio C. Reginio, Jonina Marie J. Tengco, Christian Seagan, Joel H G Tolentino, Dennis Marvin O. Santiago, Alisdair R. Fernie, Nese Sreenivasulu

**Affiliations:** a Consumer-driven Grain Quality and Nutrition Center, Rice Breeding Innovations Platform, International Rice Research Institute, Los Baños 4030, Philippines; b Institute of Food Science and Technology, College of Agriculture and Food Science, University of the Philippines Los Baños College, Laguna 4031, Philippines; c Department of Chemistry, College of Science, De La Salle University, 2401 Taft, Avenue, Manila 0922, Philippines; d Centre of Excellence in Rice Value Addition (CERVA), (IRRI)-South Asia Regional Centre (ISARC), International Rice Research Institute, Varanasi, Uttar Pradesh, India; e Department of Food Science and Chemistry, University of the Philippines Mindanao, Mintal, Tugbok District, Davao City 8000, Philippines; f Max-Planck-Institute of Molecular Plant Physiology, Am Mühlenberg 1, Potsdam-Golm, 14476, Germany

**Keywords:** Microencapsulation, Pigmented rice, Phenolics, Antioxidant, Anticancer, Bioaccessibility

## Abstract

Pigmented paddy rice is rich in a diverse array of phytochemicals that confer notable antioxidant and anticancer properties. However, the stability and bioaccessibility of these bioactive compounds present significant challenges. In this study, 542 brown (including pigmented and non-pigmented) whole grain rice samples were screened for their antioxidant components and capacity, leading to the identification of three superior cultivars: Balatinao variable purple rice, Ketan Hitam variable purple rice, and Kintuman red rice. Using response surface methodology, rice bran extracts from these cultivars were subjected to microencapsulation to stabilize the phytochemicals. Among the microencapsulated rice bran extracts (MRBEs), Ketan Hitam MRBE demonstrated significantly higher total phenolic content (TPC) and antioxidant capacity. This enhancement is likely due to the increased concentrations of isovitexin, luteolin 7-glucoside, and vitexin following microencapsulation. Furthermore, compared to non-encapsulated rice bran extracts, MRBEs exhibited significantly improved anticancer activity against HCT116 (colon) and A549 (lung) cancer cell lines (*P* < 0.05). Subsequent fractionation of the MRBE allowed for the identification of the most bioactive fractions, which contained metabolites effective against these cancer cell lines. In addition, *in vitro* bioaccessibility assays revealed a controlled release of 19 targeted phenolic compounds. This release profile was characterized by an initial increase during the gastric digestion phase, followed by a decrease in the intestinal phase. Notably, phenolic compounds such as chlorogenic acid, gallic acid, and vanillin were preserved across the three rice varieties after microencapsulation. These findings underscore the potential of MRBEs as functional food ingredients or supplements, offering improved bioaccessibility of phenolics, enhanced antioxidant properties, and promising anticancer activity. The results support the integration of rice bran extracts into the rice value chain, promoting their use in functional health applications.

## Highlights

• Screening of pigmented rice collection revealed variation in antioxidants.
• Superior MRBEs preserved specific phenolics and exhibited anticancer property.
• Metabolomics revealed MRBE phenolic compounds preserved during *in vitro* digestion.

## Graphical abstract

**Figure:**
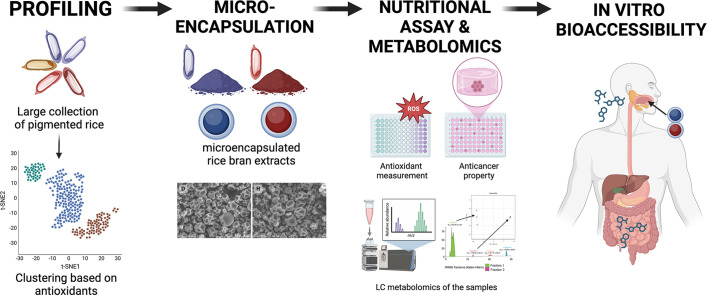

## 1 Introduction

Rice (*Oryza sativa* L.) serves as a primary staple food for a large part of the world, providing approximately 700 calories daily for about two-thirds of the global population. By 2050, the anticipated growth in population is expected to result in a 30 % increase in the demand for rice. Brown rice contains an intact outer bran layer and embryo enriched with micronutrients, fiber, and phenolic compounds. However, white rice, the form predominantly consumed worldwide, has its bran removed during the milling process. Rice bran is packed with fiber, protein, healthy fats, vitamins, and antioxidants, which can boost gut health, fight cell damage, and potentially improve heart health, blood sugar control, and cognitive function (Manzoor et al., 2023). Global rice bran production is estimated at approximately 76 million tons annually; while traditionally used as animal feed or discarded as waste, recent studies highlight its growing applications as a planting medium and as a source of bioactive compounds in both food and non-food formulations (e.g., cosmetics, pharmaceuticals, and biodegradable materials) (Tan et al., 2023). Compared with conventional brown rice bran, pigmented rice bran contains more bioactive compounds responsible for its antioxidant and anti-inflammatory properties (Tiozon et al., 2023a). Indeed, their incorporation into the human diet represents a multi-pronged approach to sustained human health. However, certain bioactive compounds derived from bran are unstable and prone to degradation during processing and storage (de Sena Andrade et al., 2023). In addition, it is crucial to note that phenolic compounds are heat-sensitive substances, and their biological properties may be affected by high temperatures upon cooking (de Sena Andrade et al., 2023). Thus, the use of effective alternatives of deploying pigmented bran is necessary to enhance their stability and ensure the efficacy of their mechanism of action with respect to functional properties.

Microencapsulation is an emerging technology that safeguards various food components or functional constituents from diverse processing conditions. A polymeric or non-polymeric material is used to envelope beneficial components, which enables their controlled release under specific conditions applied to microencapsulate fruit anthocyanins as functional food (Choudhury et al., 2021). Spray drying stands out as one of the most widely employed microencapsulation techniques due to its ability to facilitate rapid water evaporation and maintain low temperatures within the particles (Choudhury et al., 2021). Pigmented rice bran extracts have been demonstrated to contain flavonoids, anthocyanins and phenolic compounds comparable to those found in other berries. In rice, both spray-drying and freeze-drying techniques applied to microencapsulate anthocyanins with maltodextrins from glutinous variable purple rice, revealing greater stability in the spray-drying process (Laokuldilok & Kanha, 2017). Likewise, spray-drying method applied to glutinous rice starch to encapsulate anthocyanin extracts from purple rice bran (Das et al., 2019). However, significant degradation was observed during the storage process. Hence, it is imperative to identify superior rice cultivars within the gene bank that exhibit high levels of phenolic compounds with enriched flavonoids and anthocyanins and optimize the process of microencapsulation.

The health-promoting effects of phenolic compounds are associated with the endogenous content in the grain/bran of a superior variety and their bioaccessibility. Technological solutions, like encapsulation, have been shown in colonic fermentation and *in vitro* digestion studies to address bioaccessibility issues by improving absorption, solubility, and sensitivity under digestive conditions (Brodkorb et al., 2019). Despite available data from encapsulation experiments, there remains a gap in knowledge regarding encapsulated rice-bran-derived phenolic compounds for food and nutraceutical applications. This study aims to (i) develop microencapsulated phenolic-rich rice bran extracts (MRBEs) from superior rice cultivars and evaluate their nutritional and quality attributes, (ii) assess the anticancer potential of these MRBEs against HCT116 (colon) and A549 (lung) cancer cell lines, (iii) conduct a semi-purification process to identify the phenolic compounds responsible for their anticancer effects, and (iv) investigate the stability and bioaccessibility of these MRBEs through *in vitro* digestion assay, characterizing the specific phenolic compounds present at each digestion phase. The microencapsulated extracts derived from superior rice bran could be strategically positioned as functional food ingredients that could provide alternative dietary interventions to address the triple burden nutritional challenges.

## 2 Materials and methods

### 2.1 Screening large diversity set of pigmented rice for their antioxidant component and antioxidant capacity

A collection of diverse germplasm, comprising 542 paddy rice samples, including brown (*n* = 77) and various pigmented rice varieties such as purple (*n* = 38), variable-purple (*n* = 341), and red (*n* = 86), was meticulously chosen, planted, and cultivated using standard agronomic practices during the dry season of 2019 (December 2018 to May 2019) at the International Rice Research Institute (IRRI) experimental station in Los Baños, Laguna, the Philippines. The paddy rice samples were harvested and air-dried at 40−45 °C to achieve a moisture content of 14 %. Subsequently, the paddies were dehulled using a paddy rice sheller THU-35A (Satake Corporation, Hiroshima, Japan) and the brown rice were finely ground into 105 μm powder using a Mixer Mill MM400 (Germany) for all the biochemical analysis.

### 2.2 Response surface methodology (RSM) to determine the optimum extraction parameters

Parameters for the optimum extraction of phenolic compounds from rice bran were determined using the Box-Behnken Design (Supplementary Table 1). The design was used to develop robust quadratic models for a reliable prediction within the experimental domain. The effect of ethanol concentration (50–75 %), extraction time under ultrasonication (45–60 min), and extraction temperature (30–60 °C) on total phenolic content (TPC), total flavonoid content (TFC), total anthocyanin content (TAC), DPPH (2,2-diphenyl-1-picrylhydrazyl), and FRAP (ferric reducing antioxidant power) assays were evaluated (Supplementary Table 2). A total of 17 runs with 5 center point were analyzed during the measurements of TPC, TFC, TAC, DPPH, and FRAP. The desirability function was employed to minimize ethanol concentration while maximizing the responses to simplify the downstream processing, which leads to more efficient use of resources and lower production costs in large-scale production. The solution with the desirable optimization criteria has been selected to be used for the succeeding extractions of rice bran extracts (RBEs). The responses and variables were defined using the equations below:

(1) $$Y=b_{o}+b_{1}*A+b_{2}*B+b_{3}*C\left(linear\right)$$

(2) $$Y=b_{o}+b_{1}*A+b_{2}*B+b_{3}*C+b_{4}*AB+b_{5}*AC+b_{6}*BC+b_{7}*A^{2}+b_{8}*B^{2}+b_{9}*C^{2}\left(quadratic\right)$$

Y is the response
b_o_ to b_9_ are the linear, cross products, and quadratic coefficients
A, B, and C are the variables

The statistical significance of all model coefficients was determined at a 95 % confidence level using the Design-Expert software, and confirmatory runs were done right after.

### 2.3 Preparation of rice bran and rice bran extract

**Fig. 1:**
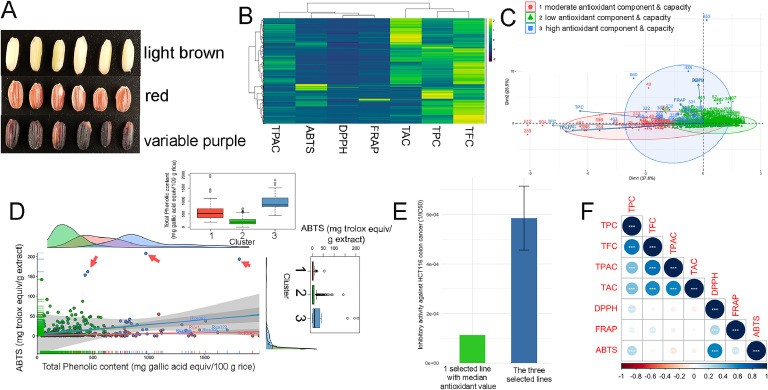
Screening large collection of brown whole grain rice for antioxidant -component and -capacity (A) comparisons of light brown, red, and variable purple rice samples used, (B) heatmap showing the variation of antioxidant component and capacity in the large diversity collection of brown rice, (C) Agglomerative nesting through Wards to cluster samples based on their antioxidant component and capacity, (D) Delineating top three samples based on TPC and ABTS, (E) inhibitory activity of colon cancer line from the top 3 brown whole grain superior variety samples versus sample from median value, (F) Correlation plot among the antioxidant traits. TPC - Total Phenolic Content; TFC - Total Flavonoid Content; TPAC - Total Proanthocyanidin Content; TAC - Total Anthocyanin Content; DPPH - 2,2-diphenyl-1-picrylhydrazyl; ABTS - 2,2′-azino-bis(3-ethylbenzothiazoline-6-sulfonic acid; FRAP - Ferric Reducing Antioxidant Power.

Approximately 200 g of brown rice from various purple and red paddy rice varieties were polished using a Grain Polisher for 100–120 s, depending on the variety. Polishing was performed until the residual bran layer was effectively removed without over polishing, to minimize starch loss. The resulting rice bran was then sieved through a no. 100 mesh (105 µm) to ensure particle size uniformity and increase the surface area-to-volume ratio for extraction (Fig. 1). The rice bran (MC = 10 to 15 %) was placed in a clean baking tray and oven-dried for 30 min at 75 °C to achieve a moisture content of 5 to 7 % and to inactivate endogenous lipase and then brought to room temperature protected from light at room temperature (Bhanger et al., 2008). Rice bran was then packed in an aluminum/polyethylene bag to prevent auto-oxidation and kept at ambient temperature until further use. The extraction of phenolic compounds was conducted using the optimum extraction conditions with the highest desirability obtained from the RSM conducted, where 50 g rice bran was added with 1000 mL of 51.244 % ethanol solution. The solution was ultrasonicated for 60 min at 60 °C and then filtered with cheesecloth. The supernatant was pooled in 5 L amber bottles. The solvent was removed using a rotary evaporator at 50 °C. The crude RBEs were kept in amber bottles at 4 °C until further analysis.

*Preparation of feed mixture.* To prepare the encapsulating material, 300 g of maltodextrin (MDDE10) and 75 g of gum arabic were dispersed in 2500 mL distilled water (40 °C) and stirred magnetically at 350 rpm for 2 h at room temperature. The mixture was then refrigerated (4 ± 1 °C) overnight for complete hydration. Subsequently, a feed solution was created by combining 2500 mL of RBE (15 % total soluble solids w/v) with the prepared hydrated encapsulating material at a 1:1 ratio and homogenized using a high-speed blender for 5 min. This dispersion of wall material and RBE was then spray-dried to obtain 750 g of dry powder. The spray drying was performed under the following conditions: inlet temperature (178 °C), outlet temperature (80 °C), blower (0.9 m³/min), and atomizing pressure (90 kPa) using a Siemens LPG5 High Speed Centrifugal Spray Dryer (Chayaratanasin et al., 2015). Samples of spray-dried material were kept in an aluminum (Al)/polyethylene (PE) bag under refrigerated condition until further use*.*

### 2.4 Characterization of spray-dried material

#### 2.4.1 Color measurements

The color characteristics of the spray-dried rice bran extract (RBE) powder were assessed using a Konica-Minolta Chroma Meter CR-410 (Konica-Minolta Sensing, Tokyo, Japan), which had been calibrated against a standard white tile. Measurements were recorded as Hunter color values: L* (lightness), a* (red-green spectrum), and b* (yellow-blue spectrum), along with chroma (C*) and hue angle (h*) following the method described by Itagi et al. (2023). Each value represents the average of three independent measurements.

#### 2.4.2 Encapsulation efficiency

Encapsulation efficiency was performed according to the previous method using the following equation (da Rosa et al., 2021):

(3) $$Mefficiency\left(\%\right)=\left(\left(M_{TPC}-M_{SPC}\right)/M_{TPC}\right)\times 100$$

where TPC means Total Phenolic Compounds and SPC means Surface Phenolic Compounds

#### 2.4.3 Moisture content and hygroscopicity

The moisture content of spray-dried powder samples was determined according to the previous method at 135 ± 2 °C using a Sartorius moisture analyzer (Sartorius MA35; Sartorius Company, Göttingen, Germany) (Cunniff & Washington, 1997). Hygroscopicity was evaluated based on the method described previously (da Rosa et al., 2021) (grams of adsorbed moisture per 100 g of dry matter).

#### 2.4.4 WSI, WAI, SC, and bulk density

Water solubility index (WSI), water absorption index (WAI), swelling capacity (SC), and bulk density were conducted using the following formula.

(4) $$Water\;Solubility\;Index\;=\;\frac{Supernatant\;dried\;weight}{Initial\;weight\;microcapsules}x\;100$$

(5) $$Water\;Absorption\;Index\;=\;\frac{Fresh\;sediment\;weight}{Initial\;weight\;microcapsules}x\;100$$

(6) $$Swelling\;Capacity\;=\;\frac{Supernant\;dried\;weight}{Initial\;weight\;microcapsules\;\left(100-WSI\right)}$$

(7) $$Bulk\;density\;=\;\frac{Weight\;of\;spray-dried\;powder\;\left(g\right)}{Volume\;of\;spray-dried\;powder\;\left(cm^{3}\right)}$$

#### 2.4.5 Morphology

The surface structure of the samples was examined using a Hitachi SU1510 Scanning Electron Microscope (SEM) (Thermo Fisher Scientific, Massachusetts, USA). Imaging was conducted at an accelerating voltage of 15 kV, with both the probe and filament currents adjusted to 70 µA. Prior to analysis, the samples were coated with a thin layer of platinum using a Hitachi MC1000 ion sputter coater, operating at 40 mA for 30 s.

### 2.5 Fourier Transform Infrared spectroscopy (FTIR)

Mid-infrared spectra of the samples were acquired using a Fourier Transform Infrared (FTIR) spectrophotometer (IRSpirit-X, Shimadzu, Kyoto, Japan) equipped with a QATR-S diamond single-reflection accessory and a zinc selenide focusing crystal. Spectral data were collected in the range of 4000 to 400 cm⁻¹, with a resolution of 4 cm⁻¹, averaging 64 scans per sample. The spectra of the samples were taken in triplicates.

### 2.6 Spectrophotometric measurements for antioxidant component and capacity

Antioxidant components, including TPC, TFC, TAC, and total proanthocyanidin content (TPAC), were assessed following established protocols (Tiozon et al., 2023a). TFC was determined using the aluminum chloride colorimetric method, while TPC was quantified via the Folin-Ciocalteu assay. TAC was measured using the pH differential technique, and TPAC was evaluated through the vanillin assay. Absorbance readings were obtained using a microplate reader (SPECTROstar Nano, BMG Labtech, Germany). Results were expressed as mg gallic acid equivalents (GAE) per 100 g for TPC, mg catechin equivalents (CE) per 100 g for both TFC and TPAC, and as cyanidin-3-O-glucoside (Cyn-3-Glu) equivalents for TAC. Antioxidant capacity tests, including DPPH, FRAP, and ABTS assays, followed methods outlined by Gulcin (2020). Trolox in ethanol (serially diluted) was used as a positive control, with a blank control also prepared. Absorbance readings for DPPH, FRAP, and ABTS were taken at 515 nm, 620 nm, and 734 nm, respectively. All antioxidant values were reported as Trolox equivalents per 100 g of brown rice (μmol TE/100 g), with each measurement based on three independent biological replicates.

### 2.7 Anticancer activity of microencapsulated superior rice bran extracts

The cytotoxic activity of MRBEs was evaluated against human colon carcinoma (HCT116) and lung adenocarcinoma (A549) cell lines, both obtained from the American Type Culture Collection (Manassas, VA, USA), using the 3-(4,5-dimethylthiozol-2-yl)-2,5-diphenyltetrazolium bromide (MTT) assay with modifications based on the method of Brotman et al. (2021). In brief, cells were cultured at 37 °C in a humidified incubator maintained at 5 % CO₂ and ∼95 % relative humidity. Once cell confluence exceeded 70 %, they were harvested via trypsinization, collected, and adjusted to a density of 50,500 cells/mL. A 198 µL aliquot of this suspension was dispensed into each well of a sterile 96-well microplate, followed by overnight incubation under the same conditions.

Subsequently, 2 µL of serially diluted MRBEs were added to designated wells, achieving final concentrations of 240, 120, 60, 30, 15, and 7.5 µg/mL. For comparison, doxorubicin at an initial concentration of 2 µg/mL was used as the positive control through two-fold serial dilutions, while DMSO served as the vehicle control. All experiments were conducted in triplicate and repeated independently three times.

After 72 h, the media in each well was discarded, and 5 mg/mL MTT solution prepared in phosphate-buffered saline (PBS) was added. Plates were incubated for an additional 4 h before adding DMSO to solubilize the formazan crystals. Absorbance was measured at 570 nm using a microplate reader. Growth inhibition was calculated using a standard formula, and the IC₅₀ values were derived from the linear portion of the dose-response curve indicating 50 % inhibition.

(8) $$\%inhibition\;=\;100\;-\frac{Abs\;extract\;-\;Abs\;blank}{Abs\;vehicle\;control\;}x\;100$$

### 2.8 Simulated *in vitro* digestion and bioaccessibility of antioxidant components and capacities and targeted bioactive compounds

Spray-dried samples of MRBEs were subjected to a static three-stage *in vitro* digestion (Brodkorb et al., 2019). This method simulates the oral, gastric, and intestinal phases of human digestion. Specific enzymes and simulated fluids on each phase were used to replicate the conditions of each digestion stage. This method provided insights into sample behavior and potential bioavailability throughout the digestive process. During digestion, samples (1 mL) were obtained at various time points during the gastric (G0: 0 min, G120: 120 min) and intestinal phases (I0: 0 min, and I120: 120 min). Aliquot samples were centrifuged at 9000 RCF for 10 min. The supernatant, comprising the soluble component and thereby representing the absorbable fraction, was collected and characterized for its antioxidant components and antioxidant capacity, as well as its specific phenolic compounds. The percent bioaccessibility of each identified bioactive compound was also determined using the following equation (Ferreira et al., 2022).

(9) $$\%Bioaccessibility=\;\frac{Final\;concentration\;at\;each\;digestion\;step}{Initial\;concentration\;before\;digestion}x\;100$$

### 2.9 Untargeted and targeted metabolomics through high-resolution mass spectrometer

The extraction and processing of the samples for metabolomic analyses followed the methodologies described in previous studies (Tiozon et al., 2023b). The Ketan Hitam rice extract was fractionated into four distinct fractions (Supplementary Fig. 1). The first and second fractions, which exhibited relatively effective inhibitory values in MTT assays, were subjected to further targeted and untargeted metabolomics analyses. The 19 phenolic metabolites were quantified using the standards with R² = 0.999 following the previous method. Mass spectra were acquired using an Orbitrap high-resolution mass spectrometer: Fourier-transform mass spectrometer (FT-MS) coupled with a linear ion trap (LTQ) Orbitrap XL (ThermoFisher Scientific, https://www.thermofisher.com). Chromatograms and mass spectra were analyzed using Chroma TOF 4.5 (Leco) and TagFinder 4.2 software. Metabolite data correlation was assessed using Expressionist Analyst 14.0.5 (Genedata, Basel, Switzerland) (https://www.genedata.com/products/expressionist). The metabolite reporting checklist is provided in Supplementary Table 4.

## 3 Results and discussion

### 3.1 Screening of large diversity pigmented rice in terms of antioxidant component and capacity

Since rice samples vary greatly in terms of nutritional properties with varied degree of pigmentation (Fig. 1A), it is imperative to screen the gene bank for the samples with high antioxidant components and capacity. A total of 542 brown rice samples were tested for their antioxidant components, such as TPC, TFC, TPAC, and TAC, and antioxidant capacities, including DPPH, FRAP, and ABTS assays. The Agglomerative Nesting (AGNES) Ward clustering technique was employed to identify three cluster groups. Superior lines with high antioxidant components and capacity represented within cluster 3 (*n* = 38) (Fig. 1B and C). Clusters 1 and 2 comprised samples with moderate and low antioxidants, respectively. A positive correlation was observed among the variables, particularly between TPC and ABTS, which aided in the identification of superior lines in terms of high antioxidant components and capacity. Across the clusters, the pigmentation of the brown rice samples varied. Buenafe et al. (2022) demonstrated significant variation in the nutritional composition of pigmented rice samples, highlighting that color alone is not a reliable indicator of nutritional value (Buenafe et al., 2022). It is noteworthy, however, that the superior cluster predominantly consisted of red and variable purple rice. The Principal Component Analysis (PCA) revealed that PC1 (37.6 % variation) is primarily influenced by TPC, while PC2 (25.5 % variation) is significantly impacted by ABTS and DPPH. In fact, TPC measures the overall phenolic compounds comprising flavonoids, anthocyanins, and proanthocyanidins (Tiozon et al., 2023a). ABTS assesses antioxidant activity through mechanisms involving hydrogen atom transfer and single-electron transfer, making it particularly suitable for evaluating phenolic antioxidants (Ilyasov et al., 2020). In this context, TPC and ABTS (Supplementary Table 5) were used to delineate three samples from Cluster 3, which comprised high antioxidant components and capacity (Fig. 1D). Consistently, these three samples delineated superior antioxidant capacity to the rest of the collection when compared between ABTS and flavonoids, anthocyanins, and proanthocyanidins (Supplementary Fig. 2). Furthermore, the three brown whole grain samples exhibited stronger inhibitory activity against HCT116 colon cancer cells compared to the sample with a median antioxidant value (Fig. 1E). The identified three lines were Balatinao variable purple rice, Ketan Hitam variable purple rice, and Kintuman red rice. Ketan Hitam originates from Indonesia, whereas Balatinao and Kintuman are traditional varieties from the Philippines. Notably, the antioxidant activity measured through FRAP assay has no significant correlations with TPC, TAC, TPAC and a weak correlation with TFC (Fig. 1F).

### 3.2 Response surface methodology (RSM) to optimize the extraction of rice bran for microencapsulation

A Box-Behnken design was employed to optimize the extraction of phenolic compounds from rice bran (Supplementary Tables 1 and 2). The objective of the RSM is to determine the extraction method that will elicit the highest TPC, TFC, TAC, DPPH scavenging activity, and FRAP among the three rice bran varieties. The developed response surface equations for predicting the percentage of responses were fitted to the linear and polynomial models (Supplementary Table 6). The results of the ANOVA analyses for the responses were found to be significant, whereas the *P*-values for TPC, TFC, TAC, DPPH scavenging activity, and FRAP were 0.0025, 0.0015, <0.0001, <0.0001, and <0.0001, respectively. TAC, DPPH, and FRAP have excellent model fits, whereas R^2^ and adjusted R^2^ values are both above 0.9, indicating the models capture the relationship between the variables and can be reliably used for prediction (Supplementary Table 7). However, the TPC and TFC were observed to have R^2^ values above 0.5 and adjusted R^2^ values below 0.7, suggesting a moderate model fit. Additionally, the *P*-value for the lack of fit tests of the responses was observed to be >0.05, which indicates that the model fits well (Supplementary Table 8). It can be surmised that the ethanol concentration, extraction temperature, and extraction time have a statistically important effect on the antioxidant component and capacity, specifically on TAC, DPPH, and FRAP, which is illustrated using response surface graphs generated based on two independent variables (Supplementary Fig. 3). Confirmatory runs show that observed means were higher in terms of TPC, TFC, TAC, and FRAP compared to the predicted values and similar in terms of the DPPH scavenging activity (Supplementary Table 9).

Similarly, the optimization of Belwal et al. (2016) suggested that extraction parameters such as solvent concentration, extraction time, and temperature may affect the accuracy quantification of phenolic and antioxidants in *Berberis asiatica* using HPLC which corroborates the current study. Under optimized conditions, one of the optimal combinations of independent variables was 51.244 % ethanol, 60 °C, and a 1 h extraction time. This combination yielded the maximum total phenolic content (TPC: 3376.67 mg GAE/100 g), total flavonoid content (TFC: 2006.29 mg CE/100 g), total anthocyanin content (TAC: 2608.47 mg C3G/100 g), DPPH scavenging activity (58.94 mg TE/100 g), and ferric reducing antioxidant power (FRAP: 312.81 mg TE/100 g). These results reflect significantly higher antioxidant components and capacity compared to the study by Irakli et al. (2018), where their optimized extraction method, using an extraction temperature of 40 °C, a 10 min extraction time, and 56 % ethanol concentration, resulted in lower TPC (378 ± 15 mg GAE/100 g) and ABTS (612 ± 24 mg TE/100 g) values.

### 3.3 Physicochemical properties of the microencapsulated superior rice bran extracts

Encapsulation emerges as a promising tool for preserving the beneficial properties of phenolic compounds while mitigating any undesirable aspects, such as bitter taste or unpleasant odor (Choudhury et al., 2021). In particular, the widely adopted spray-drying method is prevalent in the food industry due to its cost-effectiveness. Herein, the spray-drying method was employed on the three superior pigmented rice extracts selected based on their antioxidant components and capacity.

**Fig. 2:**
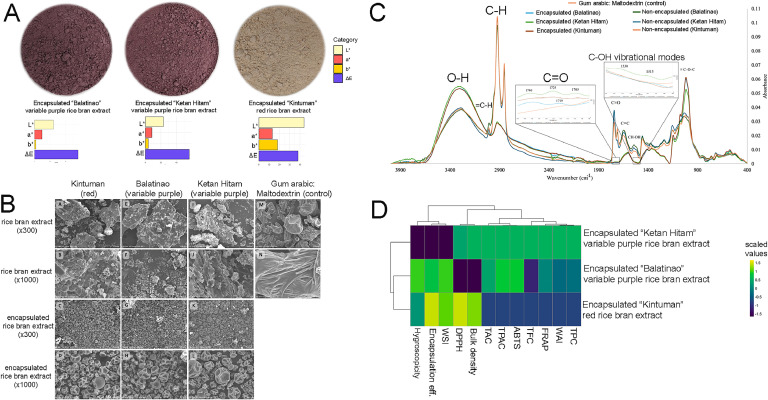
Microencapsulated rice bran extracts (A) photograph of top rice bran extracts subjected to microencapsulation and their color parameters, (B) scanning electron micrographs of microencapsulated rice bran extracts, (C) FTIR peaks of encapsulated and non-encapsulated rice bran extracts, (D) encapsulation efficiency, physicochemical properties, and antioxidant data of top encapsulated rice bran extracts. L* - Lightness; a* - green-red axis; b* - blue-yellow axis; ΔE - measure of color difference; TPC - Total Phenolic Content; TFC - Total Flavonoid Content; TPAC - Total Proanthocyanidin Content; TAC - Total Anthocyanin Content; DPPH - 2,2-diphenyl-1-picrylhydrazyl; ABTS - 2,2′-azino-bis(3-ethylbenzothiazoline-6-sulfonic acid; FRAP - Ferric Reducing Antioxidant Power; WSI - Water Solubility Index; WAI - Water Absorption Index.

The physicochemical properties of three MRBE were tested, as shown in Table 1. Fig. 2A displays the appearance of MRBE, with both Balatinao (L* = 24.21 ± 0.01; a* = 9.50 ± 0.03; b* = 1.70 ± 0.01) and Ketan Hitam (L* = 23.35 ± 1.17; a* = 8.51 ± 0.01; b* = 3.71 ± 0.02) exhibiting almost identical dark color for the two variable purple samples (Table 1). Conversely, the encapsulated red rice bran extract displays a light red powder with higher L* (43.94 ± 0.43), a* (13.18 ± 0.20), and b* (18.23 ± 0.42) values compared to the other two variable purple varieties (Table 1). The microencapsulation process generally resulted in a noticeable increase in the lightness of MRBE, attributed to the properties of the wall material used, consistent with previous findings (Millinia et al., 2024). The importance of characterizing the color of the MRBEs, other than being an indicator of high-value phytochemicals and antioxidant capacity, is that it determines consumer acceptability as this property may influence the color of the food and non-food application of the MRBEs (Dey & Nagababu, 2022).

**Table 1:** Physicochemical properties of the Kintuman, Balatinao, and Ketan Hitam RBEs and MRBEs.

Physicochemical Properties	Kintuman brown rice	Balatinao brown rice	Ketan hitam brown rice	Kintuman rice bran	Balatinao rice bran	Ketan Hitam rice bran	Kintuman rice bran extract	Balatinao rice bran extract	Ketan Hitam rice bran extract	Kintuman spray-dried rice bran extract	Balitanao spray-dried rice bran extract	Ketan Hitam spray-dried rice bran extract
L*	42.72 ± 2.04 aB	33.44 ± 0.72 bA	30.02 ± 0.09 cA	59.91 ± 2.73 aA	27.96 ± 1.23 bB	27.10 ± 0.07 bB	-	-	-	43.94 ± 0.43 aB	24.21 ± 0.01 bC	23.35 ± 1.17 bC
a*	14.21 ± 0.82 aB	8.64 ± 0.17 bB	5.10 ± 0.020 cC	21.81 ± 1.14 aA	6.85 ± 0.30 bC	5.97 ± 0.04 bB	-	-	-	13.18 ± 0.20 aB	9.50 ± 0.03 bA	8.51 ± 0.01 cA
b*	17.62 ± 1.20 aB	4.45 ± 0.06 bA	5.39 ± 0.12 bA	26.68 ± 1.36 aA	0.96 ± 0.07 bC	2.45 ± 0.03 bC	-	-	-	18.23 ± 0.42 aB	1.70 ± 0.01 cB	3.71 ± 0.02 bB
Chroma	22.63 ± 1.45 aB	9.72 ± 0.18 bA	7.38 ± 0.02 cB	34.46 ± 1.78 aA	6.92 ± 0.30 bB	6.46 ± 0.04 bC	-	-	-	22.5 ± 0.46 aB	9.66 ± 0.03 bA	9.03 ± 0.02 cA
Hue Angle	51.09 ± 0.31 aB	27.24 ± 0.20 cA	46.21 ± 0.07 bA	50.73 ± 0.05 aB	7.95 ± 0.24 cC	22.34 ± 0.09 bC	-	-	-	54.12 ± 0.22 aA	10.13 ± 0.09 cB	24.46 ± 0.05 bB
MC, % wb	-	-	-	12.50 ± 0.10 bC	12.37 ± 0.06 bB	12.37 ± 0.06 aB	98.79 ± 0.08 aA	98.39 ± 0.13bA	97.05 ± 0.09 cA	3.08 ± 0.19 aB	3.15 ± 0.42 aC	2.83 ± 0.38 aC
TS, % wb	-	-	-	87.50 ± 0.10 aB	87.63 ± 0.06 aB	86.53 ± 0.06 bB	1.21 ± 0.08 cC	1.61 ± 0.13 bC	2.95 ± 0.09 aC	96.92 ± 0.19 aA	96.85 ± 0.42 aA	97.17 ± 0.38 aA
Water Solubility Index, %	-	-	-	6.48 ± 0.00 cB	15.37 ± 0.00 aB	9.76 ± 0.00 bB	-	-	-	92.71 ± 0.00 aA	93.04 ± 0.00 aA	91.05 ± 0.00 aA
Water Absorption Index, g/g	-	-	-	0.99 ± 0.00 bA	1.00 ± 0.00 aA	0.99 ± 0.00 abA	-	-	-	0.24 ± 0.01 cB	0.32 ± 0.01 bB	0.40 ± 0.00 aB
Swelling Capacity, g/g	-	-	-	0.001 ± 0.000 cB	0.002 ± 0.000 aB	0.001 ± 0.000 bB	-	-	-	0.009 ± 0.000 aA	0.009 ± 0.001 aA	0.009 ± 0.000 aA
Encapsulation efficiency, %	-	-	-	-	-	-	96.79 ± 0.01 a	95.61 ± 0.00 a	81.08 ± 0.00 b	-	-	-
Hygroscopicity, %	-	-	-	-	-	-	-	-	-	2.00 ± 0.05 a	1.82 ± 0.24 a	1.90 ± 0.13 a
Bulk density, g/mL	-	-	-	-	-	-	-	-	-	0.42 ± 0.01 b	0.49 ± 0.01 a	0.48 ± 0.01 a

Values are mean ± standard deviation of three independent determinations (*n* = 3); Small letters denote a significant difference (*p* < 0.05) in rice bran, rice bran extract, and spray-dried rice bran extract, while capital letters denote significant differences (*p* < 0.05) within genotypes; “-” - not applicable parameters. L* - Lightness; a* - green-red axis; b* - blue-yellow axis; MC: moisture content; TS: total solubility.

Among the samples, Kintuman MRBE exhibits the highest encapsulation efficiency at 96.78 ± 0.01 %, followed by Balatinao MRBE (95.61 ± 0.00 %) and Ketan Hitam MRBE (81.08 ± 0.00 %) (Table 1). The encapsulation efficiency of the MRBEs signifies the capacity of the wall material to preserve core materials and is a crucial factor in determining the sustainability of encapsulated phenolic compounds (Cilek et al., 2012). It relies on various factors, such as the polymer concentration, solubility in the solvent, and rate of solvent removal (Jyothi et al., 2010). In addition, the composition and stability of the phenolic compounds in the samples may also contribute to varying encapsulation efficiency. At the same time, the water solubility index (WSI) of the Balatinao MRBE was found to be 93.04 ± 0.00 %, whereas Kintuman and Ketan Hitam MRBEs obtained 92.71 ± 0.00 % and 91.05 ± 0.00 %, respectively (Table 1). As expected, the WSI of the MRBEs is high, mainly due to the use of maltodextrin and gum arabic as wall materials, both of which are known for their excellent water solubility. This characteristic not only demonstrates the favorable behavior of these materials in an aqueous matrix but also aligns well with industrial and consumer preferences, enhancing their applicability in various products (Xiao et al., 2022). Additionally, maltodextrin enhances WSI by distributing the feed material, which allows for easier contact with water during the dissolving process (Xiao et al., 2022). Regarding the water absorption index (WAI), Ketan Hitam (0.40 ± 0.00 g/g) was the highest, followed by Balatinao (0.32 ± 0.01 g/g) and Kintuman (0.24 ± 0.01 g/g) MRBEs (Table 1). Concurrently, the results show that the hygroscopicity of the MRBEs ranges from 1.82 to 2.00 %, which collectively indicates that the MRBEs do not absorb a large amount of moisture from the environment (Jafari et al., 2017). Interestingly, no differences between the rice bran samples were observed in their swelling capacities (SC). A microencapsulant with higher water absorption and swelling capacities may exhibit an increased propensity to absorb water, potentially influencing release characteristics and leading to controlled release of the encapsulated phenolic compounds (Jyothi et al., 2010). However, excessively high WAI and SC can hinder the initial release or degrade phenolics. This study identified optimal values that suggest a controlled and gradual release, facilitating sustained bioaccessibility throughout the digestive tract and ensuring the optimal utilization of bioactive compounds without significant degradation (Barros & Junior, 2019). Further, variable purple MRBEs showed no significant differences in bulk density (g/mL), reflecting their high total solids content (Table 1), which is a critical factor for packaging, storage, and shelf life stability in food processing. (Nguyen et al., 2024).

SEM examinations in x300 and x1000 magnifications revealed that all microcapsules (red Kintuman - B, C; variable purple Balatino - E, F; variable purple Ketan Hitam - H, I) displayed a spherical morphology with varied diameters (average diameter of 15.8 µm) (Fig. 2B). This contrasts sharply with the non-encapsulated samples. The surfaces of all rice bran extract-encapsulated microcapsules showed distinctive irregularities, including wrinkles and depressions. These microstructural features are attributed to the conditions within the drying chamber, particularly the temperature and the size of the atomized droplets, which induced rapid water evaporation from the microcapsules (Norkaew et al., 2019). Similar morphologies were observed in maltodextrin/gum arabic microcapsules encapsulated with saffron (Rajabi et al., 2015), grape polyphenols (Tolun et al., 2016), eggplant peel (Sarabandi et al., 2019) and *Citrus reticulata* (Li et al., 2024) extracts.

FTIR spectroscopy was used to characterize the changes in the intermolecular interactions and the presence of certain compounds upon encapsulation. Vibrational peaks of the O-H and C-H groups were observed around 3307 to 3285 and 2923 cm^-1^, respectively (Fig. 2C). Meanwhile, the sharp peak centered at 1017 cm^-1^ observed from the spectra of the encapsulated extracts arises from the angular deformation of the =CH and =CH_2_ bonds (Sarabandi et al., 2019). The bands at 3007, 2977, and 2853 cm^-1^ in the spectra of the non-encapsulated samples are assigned to the asymmetric and symmetric stretching of C—H, respectively (Herath et al., 2024), while the peaks on 1708, 1645, and 1459 cm^-1^ are associated with the C=O, and C=C of the aromatic rings, and =C-O-C vibrations of the flavonoids (Herath et al., 2024). The signal around 1605 cm^-1^ indicates the C=C from the aromatic constituents of samples, while the peak at 1410.87 cm^-1^ is ascribed to the symmetric and asymmetric vibrations of the carboxylic acid O—H (Herath et al., 2024).

Interestingly, the reduction in the intensity of the peaks ascribed to the aromatic components can be attributed to the extracts having been covered with the wall material, indicating the successful encapsulation (Sarabandi et al., 2019). In addition to this, the hypsochromic peak shift from 1000 to 1017 cm^-1^ may be attributed to the increased degree of intermolecular hydrogen bonding from the interaction between the aromatic constituents and wall material upon the encapsulation (Cruz‐Molina et al., 2021). This is also further evidenced by the -O-H signal broadening and peak reduction, similar to the reports from previous studies (Cruz‐Molina et al., 2021)

### 3.4 Biochemical and targeted metabolomic profiling of the microencapsulated superior rice bran extracts

Using the optimum extraction method, analyses revealed significant variations in the antioxidant components and capacity of each RBE sample, where TPC values range from 894.31 to 1538.24 mg/100 g (Supplementary Table 9). This shows the importance of utilizing these superior pigmented rice brans in terms of their phenolic composition since they offer immense health benefits due to their high antioxidant components and capacity, which include reducing chronic disease risk and lowering inflammation throughout the body (Pisoschi et al., 2024). Ketan Hitam RBE (629.66 ± 24.97 mg Cyn-3-Glucoside/ 100 g) obtained significantly the highest value of TAC, while Kintuman RBE was significantly the highest in parameters such as TFC (817.46 ± 85.61 mg/ 100 g) and DPPH (665.46 ± 7.33 mg TE/g) values (Supplementary Table 9). However, the Balatinao RBE (1349.82 ± 1.36 mg TE/g) obtained the highest ABTS value (Supplementary Table 10). Results show that the RBEs from the selected three pigmented rice brans have diverse source of antioxidant components contributing to very high antioxidant capacity, suggesting variabilities in specific phenolic groups such as flavonoids and anthocyanin and the antioxidant capacities also implicate the differences in their mode of action and health benefits (Tabart et al., 2009).

After the microencapsulation process, Ketan Hitam MRBE obtained significantly higher TPC (230.41 ± 10.99 mg/100 g), TFC (192.69 ± 16.49 mg/ 100 g), TAC (216.36 ± 7.22 mg Cyn-3-Glucoside/ 100 g), and DPPH scavenging activity (508.57 ± 2.42 mg TE/g) compared to Kintuman and Balatinao MRBEs (Supplementary Table 10). Both variable purple rice MRBEs exhibit higher TPAC value compared to red rice Kintuman MRBE. Likewise, variable purple rice Balatinao MRBE is the highest in terms of ABTS (441.83 ± 8.27 mg TE/g), followed by the second variable purple rice Ketan Hitam MRBE (335.14 ± 6.77 mg TE/g) compared to lower levels detected in red rice Kintuman MRBE (278.48 ± 46.34 mg TE/g) (Supplementary Table 9). The FRAP assay revealed that Kintuman (86.19 ± 3.20 mg TE/g) and Ketan Hitam (88.95 ± 1.21 mg TE/g) have higher antioxidants compared with Balatinao (79.62 ± 2.84 mg TE/g). The varying antioxidant capacity of the Balatinao MRBE can be attributed to the endogenous variation in bioactive compounds and the nature of the phenolic compounds retained after spray-drying (dos Santos Leal et al., 2024). Generally, the microencapsulation process has caused some reductions in the antioxidant components and capacities in all three MRBEs compared to their RBE counterpart, which corroborates the reduction in the phenolic-related FTIR peaks after the microencapsulation process (Fig. 2C). The slight degradation observed can be attributed to the increase in temperature during the microencapsulation process. Similarly, Fereira et al. (2022) observed a slight reduction in the antioxidant component and capacity of the Tucuma coproducts upon microencapsulation. However, the TPC, TFC, and TAC values obtained from the MRBEs are still in high levels, indicating their functionality in improving health. Additionally, antioxidant capacities such as high DPPH and ABTS scavenging activities suggest that the samples still have a huge potential to neutralize free radicals, offering cellular protection.

To delve deeper into the phenolic concentrations of the MRBEs and RBEs, 19 phenolic compounds were quantified across the samples (Supplementary Table 9). Balatinao and Ketan Hitam RBEs possessed higher amounts of various phenolic compounds, flavonoids and small amount of flavones, specifically isovitexin, luteolin 7-glucoside, and vitexin. Flavones are a subgroup of flavonoids that are known to have anticancer, antioxidant, anti-inflammatory, and antiviral properties (Tiozon et al., 2023a), which aligns with the high TFC and ABTS scavenging activity of Balatinao RBE. Kintuman and Balatinao RBEs were found to have higher caffeic acid, epicatechin, and p-coumaric acid than the Ketan Hitam RBE, which agrees with their corresponding TPC values. Caffeic acid, epicatechin, and *p*-coumaric acid are compounds commonly found in coffee and tea that have high antioxidant, antibacterial, and anti-inflammatory properties (Stojkovic et al., 2013). Additionally, compared to other RBEs, Balatinao obtained higher quantities of ellagic acid (2.967 ± 0.310 mg/100 g), gallic acid (0.262 ± 0.002 mg/100 g), naringenin (0.250 ± 0.005 mg/100 g), quercetin (6.243 ± 0.370 mg/100 g), and sinapic acid (0.907 ± 0.043 mg/100 g) showing its diverse and rich phenolic and flavonoid content which can also be attributed on its TPC, and ABTS scavenging activity. Kintuman RBE, on the other hand, was the highest in trans-ferulic acid (1.642 ± 0.121 mg/100 g), and Ketan Hitam RBE has the highest petunidin 3-glucoside chloride (0.099 ± 0.017 mg/100 g), and rutin hydrate (0.152 ± 0.053 mg/100 g).

**Fig. 3:**
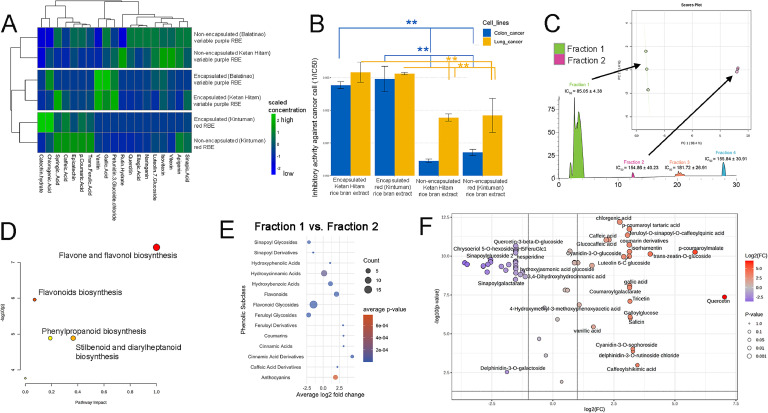
Phenolic and antioxidants of microencapsulated rice bran extracts. (A) Phenolic profile of non-encapsulated and encapsulated rice bran extracts. (B) Inhibitory activity of microencapsulated variable purple and red rice bran extracts against colon and lung cancer cell lines. (C) Semi-purification and fractionation of Ketan Hitam microencapsulated variable purple rice extracts and principal component analysis showing the difference of fractions 1 and 2 based on the metabolites in the principal components (PC 1 and PC2). (D) Pathway impact analysis of fractions 1 and 2 of Ketan Hitam MRBE, (E) subclass differentiation between fractions 1 and 2 demonstrating the accumulation of phenolic acids and flavonoids in the former and flavonoid glycosides in the latter. (F) Volcano plot showing the differentially accumulated metabolites of fractions 1 and 2 (right portion – upregulated, left portion – downregulated for fraction 1).

It was also observed that spray drying had a significant impact on the phenolic content of the MRBE samples. While the majority of these compounds decreased after the drying process, while some of the compounds, such as apigenin and rutin hydrate, were undetectable. Similar to previous studies, the TPC and antioxidant capacity of spray-dried powders from fruits and vegetables were decreased drastically, but the thermal process greatly helped against the degradation of these compounds during storage (Shoukat et al., 2024). Although both variable purple rice (Balatinao and Ketan Hitam) MRBEs exhibit higher phenolic compound and flavonoids in the microencapsulated samples, Ketan Hitam MRBE obtained the highest epicatechin (0.494 ± 0.045 mg/100 g), snapic acid (0.524 ± 0.039 mg/100 g) trans-ferulic acid (0.532 ± 0.042 mg/100 g), and vanillin (0.513 ± 0.077 mg/100 g) content compared to the other MRBEs (Supplementary Table 9), which can also be reflected in its high TPC, TAC, TPAC, FRAP, and DPPH scavenging activity (Fig. 2D). This indicates that phenolic compounds found in Ketan Hitam RBE are more stable compared to Balatinao and Kintuman RBEs when subjected to high-temperature processing such as spray drying. In red rice MRBE flavnoids such as catechin (1.080 ± 0.071 mg/100 g), epicatechin (0.476 ± 0.048 mg/100 g), and phenolic compounds such as p-Coumaric Acid (0.468 ± 0.055 mg/100 g) and trans ferulic acid (0.565 ± 0.008 mg/100 g) are being retained at higher concentrations with nearly 50 % in comparison to RBEs (Supplementary Table 10, Fig. 3A). The clustered heatmap (Fig. 3A) of the non-encapsulated and encapsulated RBE revealed that red and variable purple rice retained different set of bioactives upon microencapsulation. The microencapsulated variable purple RBEs (i.e., Ketan Hitam and Balatinao) clustered together, indicating that microencapsulation has a more prominent effect on the phenolic profile of variable purple rice compared to red rice. Previous findings reported variations in the stability of rice genotypes regarding total phenolic content, ferulic acid, and antioxidant activities (Kunnam et al., 2023). Furthermore, the stability of the phenolic compounds is also greatly influenced by the coating material (Rodrigues Vieira et al., 2024). For instance, a combination of maltodextrin, gum arabic, and whey protein retained the majority of anthocyanins, while pure gum arabic retained only selected phenolic compounds (Norkaew et al., 2019). In the present study, the combination of maltodextrin and gum arabic increased the levels of chlorogenic acid for both variable purple and red rice bran extracts. In concurrence, previous studies observed that phenolic compounds such as rutin and apigenin 7-glucoside from mahaleb cherry increased after thermal processing at 130 °C (Ghafoor et al., 2019). It can be inferred that the heat and pressure potentially have liberated bound phenolics, making them measurable and leading to an apparent increase. In addition, factors like drying temperature and the RBE's initial phenolic composition itself can influence whether an increase or a decrease in the phenolic compounds can be observed. Concurrently, de Sena Andrade et al. (2023) demonstrated that phenolic acids, such as gallic acid, are retained after microencapsulation. The present study highlighted the retention of various phenolic compounds and flavonoids upon encapsulation between red and variable purple rice bran. This finding is of special interest to the food industry because of their ability to inhibit oxidation and rancidity in oils and fats, attributed to their free radical scavenging and antioxidant properties.

### 3.5 Anti-cancer properties of the microencapsulated superior rice bran extracts

The inhibitory activity of MRBEs against HCT116 (colon cancer) and A549 (lung cancer) cell lines was assessed. Considering Ketan Hitam's superior antioxidant component and capacity, it was specifically evaluated against these cancer cell lines alongside the red Kintuman MRBE. The results revealed that encapsulated rice bran extracts exhibited significantly higher anti-cancer activity compared to their non-encapsulated counterparts (*P* < 0.01) (Fig. 3B, Supplementary Table 11). Remarkably, the microencapsulation process yielded the most pronounced anti-cancer effects against the HCT116 colon cancer cell line and A549 lung cancer cell line. Furthermore, among the MRBEs, the red Kintuman MRBE showed significantly stronger inhibitory effects against the colon cancer cell line (1/IC50 = 0.0029 ± 3.8 × 10⁻⁴ mL/µg), while the Ketan Hitam MRBE demonstrated more potent activity against the A549 lung cancer cell line (1/IC50 = 0.00317 ± 3.1 × 10⁻⁴ mL/µg). Additionally, the pigmented RBEs demonstrated no nephrotoxicity, indicating that they are safe for consumption (Supplementary Table 12). Rahaiee et al. (2020) provided a comprehensive description of how encapsulation serves as a protective mechanism for phenolic compounds, safeguarding them against deteriorating factors such as high temperature and oxygen, ultimately leading to enhanced anti-cancer properties (Rahaiee et al., 2020).

**Fig. 4:**
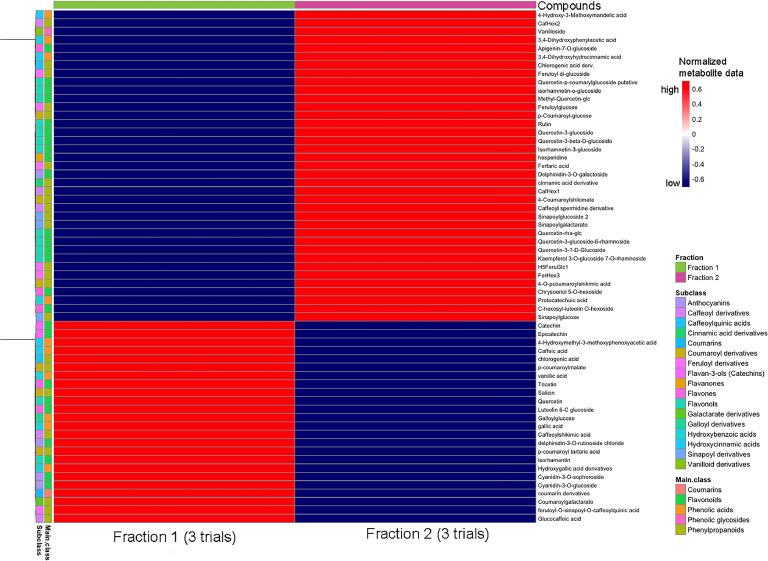
Differentially accumulated metabolites in fractions 1 and 2 of Ketan Hitam, highlighting a greater diversity of phenolic classes in fraction 1.

To pinpoint the compounds responsible for the anticancer bioactivity, the Ketan Hitam variable purple MRBE was semi-purified into four distinct fractions (Fig. 3C). The first and second fractions displayed the highest bioactivity against the HCT116 cancer cell line and were subsequently subjected to metabolomic profiling to identify the specific compounds potentially responsible for the inhibitory effects on cancer cells. The first fraction, in particular, demonstrated more effective inhibition against cancer cell lines. PCA (Fig. 3C) and a heatmap (Supplementary Fig. 4) highlighted distinct metabolite compositions between fractions 1 and 2. Further analysis revealed differences in flavonoid pathways, particularly in flavone and flavonol biosynthesis (Fig. 3D). Subclass-specific profiling (Fig. 3E) revealed that fraction 1 was enriched with flavonoids, hydroxybenzoic acids, anthocyanins, and phenolic acids, while fraction 2 exhibited higher levels of glycosides derived from feruloyl, sinapoyl, and flavonoid compounds. A volcano plot (Fig. 3F) identified specific metabolites accumulating in the two fractions. Fraction 1 contained anthocyanins such as cyanidin-3-O-glucoside and cyanidin-3-O-sophoroside, phenolic acids including vanillic acid, gallic acid, and caffeic acid, as well as other flavonoids such as catechin, epicatechin, quercetin, tricetin, and isorhamnetin, all of which significantly contribute to its observed bioactivity as shown on Fig. 4. On the other hand, fraction 2 was predominantly composed of flavonoid glycosides, including luteolin hexoside, feruloyl diglucoside, and sinapoylglucoside. It can be concluded that phenolic acids, their derivatives, and anthocyanins play a pivotal role in the enhanced anti-cancer activity, as evidenced by their higher composition and concentration in fraction 1. Consistent with this feature, microencapsulation of polyphenols from pomegranate elicited enhanced inhibitory activity against A549 and human gastric cancer cell line AGS (Fathi et al., 2022). Polyphenols inhibit cell proliferation by modulating key signaling pathways, including Erk1/2, CDK, and PI3K/Akt (Liu et al., 2023). Additionally, they can enhance intrinsic defense mechanisms by activating enzymes such as superoxide dismutase and glutathione peroxidase. This finding underscores the importance of increased levels of petunidin-3-glucoside, vanillin, and phenolic acids such as syringic acid and gallic acid for anti-cancer activities. Indeed, meta-analyses have established a robust association between the consumption of foods enriched with anthocyanins, such as petunidin-3-glucoside, and a significantly reduced risk of cancer (Neyestani et al., 2023; Wang et al., 2019). Moreover, phenolic acids, such as syringic and gallic acids, are recognized for their ability to suppress cancer cell proliferation, reduce inflammation, and induce apoptosis by upregulating mTOR via the AKT signaling pathway (Pei et al., 2021).

### 3.6 Bioaccessibility of the microencapsulated superior rice bran extracts

**Fig. 5:**
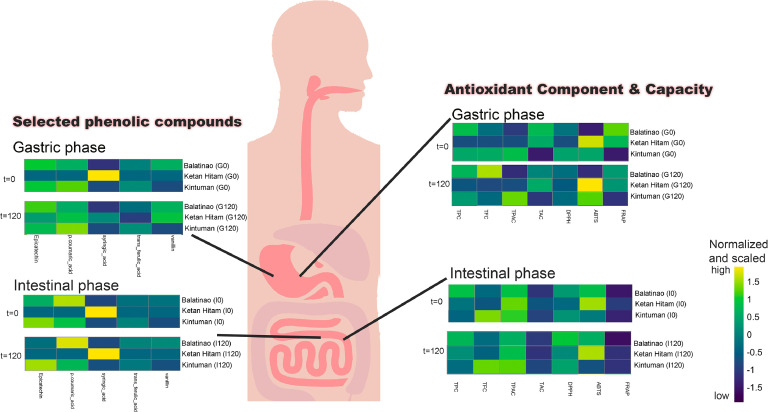
The *in vitro* bioaccessibility of microencapsulated rice bran extracts showing the phenolic profile and antioxidant activity in the gastric and intestinal phases. TPC - Total Phenolic Content; TFC - Total Flavonoid Content; TPAC - Total Proanthocyanidin Content; TAC - Total Anthocyanin Content; DPPH - 2,2-diphenyl-1-picrylhydrazyl; ABTS - 2,2′-azino-bis(3-ethylbenzothiazoline-6-sulfonic acid; FRAP - Ferric Reducing Antioxidant Power.

Bioaccessibility denotes the quantity of a compound liberated within the gastrointestinal tract, rendering it available for absorption. Hence, the effect of *in vitro* digestion on the antioxidant components and capacities of the MRBEs was observed. Generally, the majority of the antioxidant components and capacities of MRBEs were observed to increase from the gastric up to the intestinal phase (Table 2). After digestion (I_120_), TPC (89.52 ± 3.89 %), and TAC (48.47 ± 5.64 %) of variable purple rice Balatinao MRBE were observed to be higher compared to other MRBEs and the other variable purple rice Ketan Hitam has high TAC levels. However, in terms of TFC (95.46 ± 2.64 %) and TPAC (96.65 ± 0.86 %), red Kintuman MRBE was observed to be more bioaccessible (Fig. 5). Hence, high bioaccessibilities for TPC, TFC, TAC, and TPAC were observed on the MRBEs, but not all of the antioxidant components are readily available for absorption.

**Table 2:** Bioaccessibility, antioxidant components, and capacities of Kintuman, Balatinao, and Ketan Hitam MRBEs at different *in vitro* digestion phases.

Phytochemical and Antioxidant Assays	Balatinao									
	Initial	% BA	G0	% BA	G120	%BA	I0	%BA	I120	%BA
TPC, mg/100 g	202.53 ± 1.43 a	100.00 a	130.28 ± 9.61 dA	64.32 ± 4.74 dA	145.70 ± 3.49 cA	71.94 ± 1.73 cA	179.17 ± 10.82 bA	88.46 ± 5.34 bA	181.3 ± 7.88 bA	89.52 ± 3.89 bA
TFC, mg/ 100 g	176.69 ± 4.17 a	100.00 a	74.26 ± 10.09 dA	42.00 ± 5.71 dA	122.48 ± 1.2 cA	69.28 ± 0.68 cA	122.93 ± 3.46 cB	69.54 ± 1.96 cB	130.48 ± 3.24 bB	73.80 ± 1.83 bB
TPAC, mg/100 g	14.97 ± 1.51 a	100.00 a	9.40 ± 0.74 bA	62.81 ± 4.92 cA	9.72 ± 0.68 bA	64.91 ± 4.54 cB	13.54 ± 0.82 aA	90.47 ± 5.5 bA	13.57 ± 0.37 aA	90.63 ± 2.50 bB
TAC, mg Cyn-3-Glucoside/ 100 g	151.35 ± 6.85 a	100.00 a	34.81 ± 4.59 eA	23.00 ± 3.03 eA	44.71 ± 1.33 dA	29.54 ± 0.88 dA	61.78 ± 3.73 cA	40.82 ± 2.46 cA	73.36 ± 8.54 bA	48.47 ± 5.64 bA
DPPH, mg TE/g	402.15 ± 3.09 a	100.00 a	171.64 ± 11.84 eB	42.68 ± 2.94 eA	203.22 ± 14.34 dB	50.53 ± 3.57 dA	312.28 ± 29.01 cB	77.65 ± 7.21 cA	354.63 ± 16.95 bA	88.18 ± 4.21 bA
ABTS, mg TE/g	441.83 ± 8.27 a	100.00 a	178.09 ± 5.84 dA	40.31 ± 1.32 dC	203.69 ± 0.49 cA	46.10 ± 0.11 cC	375.08 ± 11.35 bA	84.89 ± 2.57 bA	377.30 ± 2.98 bA	85.39 ± 0.67 bB
FRAP, mg TE/g	79.62 ± 2.84 a	100.00 a	41.24 ± 1.96 cA	51.80 ± 2.46 cA	44.48 ± 1.73 bcA	55.87 ± 2.18 bA	44.54 ± 3.13 bA	55.94 ± 3.93 bA	47.04 ± 2.49 bA	59.08 ± 3.13 bA

Phytochemical and Antioxidant Assays	Kintuman									
	Initial	% BA	G0	%BA	G120	%BA	I0	%BA	I120	%BA
TPC, mg/100 g	191.04 ± 2.11 a	100.00 a	114.49 ± 4.97 cB	59.93 ± 2.60 cB	132.35 ± 7.76 bB	69.28 ± 4.06 bA	136.85 ± 16.75 bC	71.63 ± 8.77 bB	143.82 ± 13.78 bB	75.28 ± 7.21 bB
TFC, mg/ 100 g	182.60 ± 1.34 a	100.00 a	79.66 ± 0.71 cA	43.62 ± 0.39 eA	84.83 ± 3.17 cB	46.46 ± 1.74 dB	167.93 ± 4.17 bA	91.97 ± 2.29 cA	174.31 ± 4.81 bA	95.46 ± 2.64 bA
TPAC, mg/100 g	11.48 ± 0.03 a	100.00 a	7.76 ± 0.65 cB	67.58 ± 5.69 cB	9.52 ± 0.97 bA	82.92 ± 8.49 bA	10.60 ± 0.17 aB	92.34 ± 1.47 aA	11.10 ± 0.10 aB	96.65 ± 0.86 aA
TAC, mg Cyn-3-Glucoside/ 100 g	48.19 ± 6.84 a	100.00 a	0.24 ± 0.09 dC	0.50 ± 0.19 eC	1.60 ± 0.26 dC	3.33 ± 0.54 dC	12.78 ± 0.58 cC	26.52 ± 1.21 cB	15.30 ± 0.98 bC	31.75 ± 2.02 bB
DPPH, mg TE/g	490.76 ± 4.33 a	100.00 a	212.71 ± 5.63 cA	43.34 ± 1.15 cA	231.27 ± 1.21 cA	47.12 ± 0.25 cB	351.39 ± 40.90 bA	71.60 ± 8.33 bA	389.12 ± 56.41 bA	79.29 ± 11.49 bAB
ABTS, mg TE/g	278.48 ± 46.34 a	100.00 a	135.10 ± 11.03 cB	48.51 ± 3.96 dB	201.46 ± 2.72 bB	72.34 ± 0.98 cA	191.70 ± 11.19 bC	68.84 ± 4.02 bcB	171.70 ± 41.33 bC	61.66 ± 14.84 bC
FRAP, mg TE/g	86.19 ± 3.2 a	100.00 a	28.76 ± 0.89 dC	33.37 ± 1.03 eC	29.64 ± 0.19 dC	34.39 ± 0.22 dC	36.98 ± 0.33 cC	42.90 ± 0.38 cB	41.98 ± 0.44 bB	48.70 ± 0.51 bB

Phytochemical and Antioxidant Assays	Ketan Hitam									
	Initial	% BA	G0	%BA	G120	%BA	I0	%BA	I120	%BA
TPC, mg/100 g	230.41 ± 10.99 a	100.00 a	123.17 ± 7.61 dAB	53.46 ± 3.30 dC	129.94 ± 4.96 dB	56.39 ± 2.15 dB	162.32 ± 3.95 cB	70.45 ± 1.71 cB	186.96 ± 19.22 bA	81.14 ± 8.34 bB
TFC, mg/ 100 g	192.69 ± 16.49 a	100.00 a	66.08 ± 2.39 dB	34.29 ± 1.24 dB	71.77 ± 1.04 cdC	37.25 ± 0.54 cdC	99.23 ± 18.11 bcC	51.50 ± 9.40 bcC	122.07 ± 38.24 bB	63.35 ± 19.85 bB
TPAC, mg/100 g	15.24 ± 0.10 a	100.00 a	9.34 ± 1.66 bA	61.30 ± 10.89 bA	9.66 ± 1.60 bA	63.38 ± 10.51 bB	13.71 ± 0.60 aA	89.95 ± 3.93 aA	13.82 ± 0.56 aA	90.71 ± 3.66 aB
TAC, mg Cyn-3-Glucoside/ 100 g	216.36 ± 7.22 a	100.00 a	26.56 ± 1.14 dB	12.28 ± 0.53 eB	42.55 ± 1.30 cB	19.66 ± 0.6 dB	56.72 ± 1.62 bB	26.22 ± 0.75 cB	59.99 ± 2.64 bB	27.73 ± 1.22 bB
DPPH, mg TE/g	508.57 ± 2.42 a	100.00 a	176.01 ± 14.95 dB	34.61 ± 2.94 dB	199.06 ± 1.68 dB	39.14 ± 0.33 dC	285.77 ± 26.65 cB	56.19 ± 5.24 cB	369.12 ± 35.46 bA	72.58 ± 6.97 bB
ABTS, mg TE/g	335.14 ± 6.77 a	100.00 a	179.40 ± 7.44 eA	53.53 ± 2.22 eA	203.62 ± 1.39 dA	60.76 ± 0.41 dB	294.18 ± 2.6 cB	87.78 ± 0.78 cA	323.59 ± 1.54 bB	96.55 ± 0.46 bA
FRAP, mg TE/g	88.95 ± 1.21 a	100.00 a	37.78 ± 2.19 cB	42.47 ± 2.46 cB	39.32 ± 2.17 cB	44.21 ± 2.44 cB	39.59 ± 1.94 cB	44.50 ± 2.18 cB	45.51 ± 3.70 bA	51.16 ± 4.15 bA

**Abbreviations:** TPC - Total Phenolic Content; TFC - Total Flavonoid Content; TPAC - Total Proanthocyanidin Content; TAC - Total Anthocyanin Content; DPPH - 2,2-diphenyl-1-picrylhydrazyl; ABTS - 2,2′-azino-bis(3-ethylbenzothiazoline-6-sulfonic acid; FRAP - Ferric Reducing Antioxidant Power; WSI - Water Solubility Index; gastric (G0: 0 minutes, G120: 120 minutes) and intestinal phases (I0: 0 minutes, and I120: 120 minutes); BA: bioaccessibility.

Varying antioxidant capacities were also observed at each phase of digestion, whereas the DPPH scavenging activity of the MRBEs ranges from 34.61 to 88.18 % compared to their corresponding initial result (undigested). Meanwhile, the ABTS scavenging activity and FRAP of the MRBEs range from 40.31 to 96.55 %, and 33.37 to 59.08 %, respectively (Table 2). It is possible that the digestive process affected the antioxidant capacities of the MRBEs, whereas possible interactions with the enzymes and pH conditions during gastric and intestinal phases have occurred, causing these inconsistencies. Similarly, Cavia et al. (2023), observed varying antioxidant capacities between undigested, gastro-intestinal digested, and oral and gastro-intestinal digested cider samples (Cavia et al., 2023).

The antioxidant components and capacities at different phases of digestion were also compared. At the start and the end of the gastric phase (G_0_ and G_120_), most of the results for Balatinao and Kintuman MRBEs were observed to be significantly different. This means that the rate of antioxidant release at various time points is directly correlated with time during the gastric phase. However, during the intestinal phase (I_0_ and I_120_), similar values were observed in terms of TPC, TPAC, and ABTS on the Balatinao and Kintuman MRBEs. The release of the phenolic compounds during the intestinal phase or at neutral pH might have been the cause of these observations in the Balatinao and Kintuman MRBEs. Conversely, the majority of the antioxidant components and capacities in the Ketan Hitam MRBE were found to be similar during G0 and G120, and the TPC, TAC, DPPH, ABTS, and FRAP values were significantly different from each other during I_0_ and I_120_. The nature of the phenolic content in each rice variety might influence how they are released and contribute to antioxidant activity. For instance, Balatinao and Kintuman might have phenols more readily released at the gastric phase. At the same time, those in Ketan Hitam might be more stable during the gastric phase but release their antioxidant properties later in the intestinal phase. However, no significant differences in the TFC bioaccessibility in the Balatinao MRBE were observed during the G_120_ and I_0_, parallel to the TPC and ABTS values of the Kintuman MRBE. The TFC values of the Ketan Hitam MRBE were found to be similar during the G120 and I0 phases. Further investigation into the chemical kinetics would be needed to see if these trends continue or if further breakdown and absorption alters the bioaccessibility of these antioxidant components and capacities.

Determining the percent bioaccessibility (%B) of specific bioactive phenolic compounds plays a crucial role in influencing particular metabolic processes, which can contribute to improved overall health. Fig. 5 illustrates the %B of MRBEs, highlighting 19 bioactive phenolic compounds, with epicatechin, p-coumaric acid, and trans-ferulic acid consistently identified across all experimental treatments. Generally, there is an increase in the amount of phenolics released from MRBEs during gastric digestion and a significant decline in the intestinal phase. Most of the polyphenols were released during the gastric stage, highlighting the stomach's key role in polyphenol release, driven by the synergistic effects of pepsin enzymes and low pH (Dominguez-Avila et al., 2017). This acidic environment is conducive to preserving the structural integrity of phenolic compounds. The transition of the gastric product from pH 3 to 7, facilitated by intestinal enzymes and the high protein and other macromolecules found in rice bran extract, could potentially lead to the formation of water-soluble mixed micelles and the micellization of flavonoids. These processes may contribute to the observed low bioaccessibility during the intestinal phase (Caicedo-Lopez et al., 2019). In addition, certain phenolic compounds exhibit a strong pH dependency. Studies demonstrate that these phenolics maintain stability in acidic environments; however, they undergo hydration, ring fission, and the formation of ionized chalcones under neutral pH, leading to reduced levels during intestinal digestion (Flores et al., 2015).

It is evident that specific phenolic compounds exhibited varying levels of susceptibility during *in vitro* digestion. Previous research studies have reported the stability of epicatechin (Toro-Uribe et al., 2019), as well as p-coumaric and ferulic acids (Drawbridge et al., 2021), throughout the entire process. Furthermore, bioaccessibility exceeding 100 % has been observed for certain phenolic compounds after *in vitro* digestion (Drawbridge et al., 2021). This phenomenon can be attributed to the enzymatic breakdown of bound phenolics, particularly the disruption of interactions between phenolics and proteins, thereby enhancing their release and availability for absorption. Findings from this study revealed that microencapsulation elicits varying effects on the %B of phenolics depending on the food matrix it is applied. Specifically, microencapsulation of red rice bran extract significantly enhances the %B of epicatechin post-digestion. Concurrently, the *in vitro* digestion of encapsulated blueberry extracts steady phenolic release throughout digestion (Flores et al., 2015). Conversely, in the case of variable purple MRBEs, there is a marked decrease in %B of phenolic compounds with encapsulation. Several factors are known to affect the stability and bioaccessibility of phenolic compounds during gastrointestinal digestion, such as the presence and action of enzymes and bile salts, and some physicochemical factors including temperature, pH, and ionic strength (Minekus et al., 2014). The food matrix can also adversely affect bioaccessibility since phenolic compounds are capable of binding with macromolecules, including proteins, starch, dietary fiber, and lipids, which results in reduced amounts of polyphenols in its free accessible form. (Jakobek, 2015).

## 4 Conclusion

MRBEs of three superior rice cultivars—Ketan Hitam and Balatinao (variable purple rice) and Kintuman (red rice) subjected to microencapsulation via spray drying with a maltodextrin-gum arabic matrix produced supplements with desirable properties revealed including high encapsulation, good water solubility, and low hygroscopicity. Among the MRBEs, Ketan Hitam variable purple stood out for its superior antioxidant and anticancer properties, retaining high levels of bioactives post-encapsulation, including epicatechin, sinapic acid, trans-ferulic acid, and vanillin. It also demonstrated high TPC, TAC, and DPPH scavenging activity. Fractionation of Ketan Hitam MRBE revealed that fraction 1, rich in anthocyanins, phenolic acids, and flavonoids, exhibited potent anticancer activity, particularly against lung cancer (A549) cells (1/IC50: 0.00317 ± 3.1 × 10⁻⁴ mL/µg) and colon cancer (HCT116). Meanwhile, Kintuman (red) MRBE maintained high levels of flavonoids and phenolic acids, such as catechin, epicatechin, p-coumaric acid, and trans-ferulic acid which contributed to notable anticancer activity, particularly against HCT116 cells.

*In vitro* digestion studies revealed a distinct pattern in the release of phenolic compounds from MRBEs. Gastric digestion significantly enhanced the release of most polyphenols, likely due to the synergistic effects of pepsin and the acidic environment, whereas the intestinal phase led to a decline. Notably, the bioaccessibility of specific phenolics, including epicatechin, *p*-coumaric acid, and *trans*-ferulic acid, varied among MRBEs and across digestion stages, highlighting the substantial diversity of antioxidants in the brown whole grain rice collection. Furthermore, this study underscores the potential of MRBEs as functional food ingredients, offering dual benefits for health and agriculture. By improving the stability, bioaccessibility, and bioactivity of phenolic compounds, MRBEs present an opportunity for value addition, transforming rice bran—a traditionally underutilized byproduct—into high-value nutritional products for both consumers and farmers. These extracts, rich in antioxidants and anti-cancer properties, offer an innovative approach to addressing diet-related non-communicable diseases, contributing to improved public health while supporting sustainable agricultural practices and economic opportunities for rice producers.

## Ethical statement – studies in humans and animals

This study did not employ studies in humans and animals.

## CRediT authorship contribution statement

**Rhowell Navarro Tiozon:** Writing – original draft, Visualization, Methodology, Formal analysis, Data curation, Conceptualization. **Glenn Vincent P. Ong:** Writing – original draft, Methodology, Formal analysis. **Kristel June D. Sartagoda:** Writing – original draft, Formal analysis, Data curation, Conceptualization. **Sheba Mae M. Duque:** Writing – review & editing, Methodology. **Saleh Alseekh:** Writing – review & editing, Methodology. **Aldrin P. Bonto:** Writing – original draft, Methodology. **Shem Gempesaw:** Writing – review & editing, Methodology, Formal analysis. **Vipin Pratap:** Writing – review & editing, Methodology. **Florencio C. Reginio:** Writing – review & editing, Methodology. **Jonina Marie J. Tengco:** Writing – review & editing, Methodology. **Christian Seagan:** Writing – review & editing, Methodology. **Joel H G Tolentino:** Writing – review & editing, Formal analysis. **Dennis Marvin O. Santiago:** Writing – review & editing, Methodology. **Alisdair R. Fernie:** Writing – review & editing, Supervision, Project administration, Funding acquisition, Formal analysis. **Nese Sreenivasulu:** Writing – review & editing, Supervision, Project administration, Investigation, Funding acquisition, Formal analysis, Conceptualization.

## Declaration of competing interest

The authors declare that they have no known competing financial interests or personal relationships that could have appeared to influence the work reported in this paper.

## Data Availability

Data will be made available on request.

## Appendix Supplementary materials

Image, application 1

Image, application 2
