# Supplementary material for: Optimization of phenolic extraction method and in vitro bioaccessibility of microencapsulated pigmented rice bran extracts and their antioxidant and anticancer properties
Source: Food Hydrocoll Health. 2025 Jun;7:None. doi: 10.1016/j.fhfh.2025.100221 (PMC12167174; doi:10.1016/j.fhfh.2025.100221)
Supplement: Supplementary file 1 [file mmc1.docx]

**Supplementary Figure 1**. Chromatogram of encapsulated black bran sample at 221 nm. The HPLC parameters are 7.1 ml/min flow rate and 473 uL injection volume (10 mg/ml sample concentration). The sample is separated using a 10 mm x 250 mm C18 column. Total sample purified is 56.76 mg


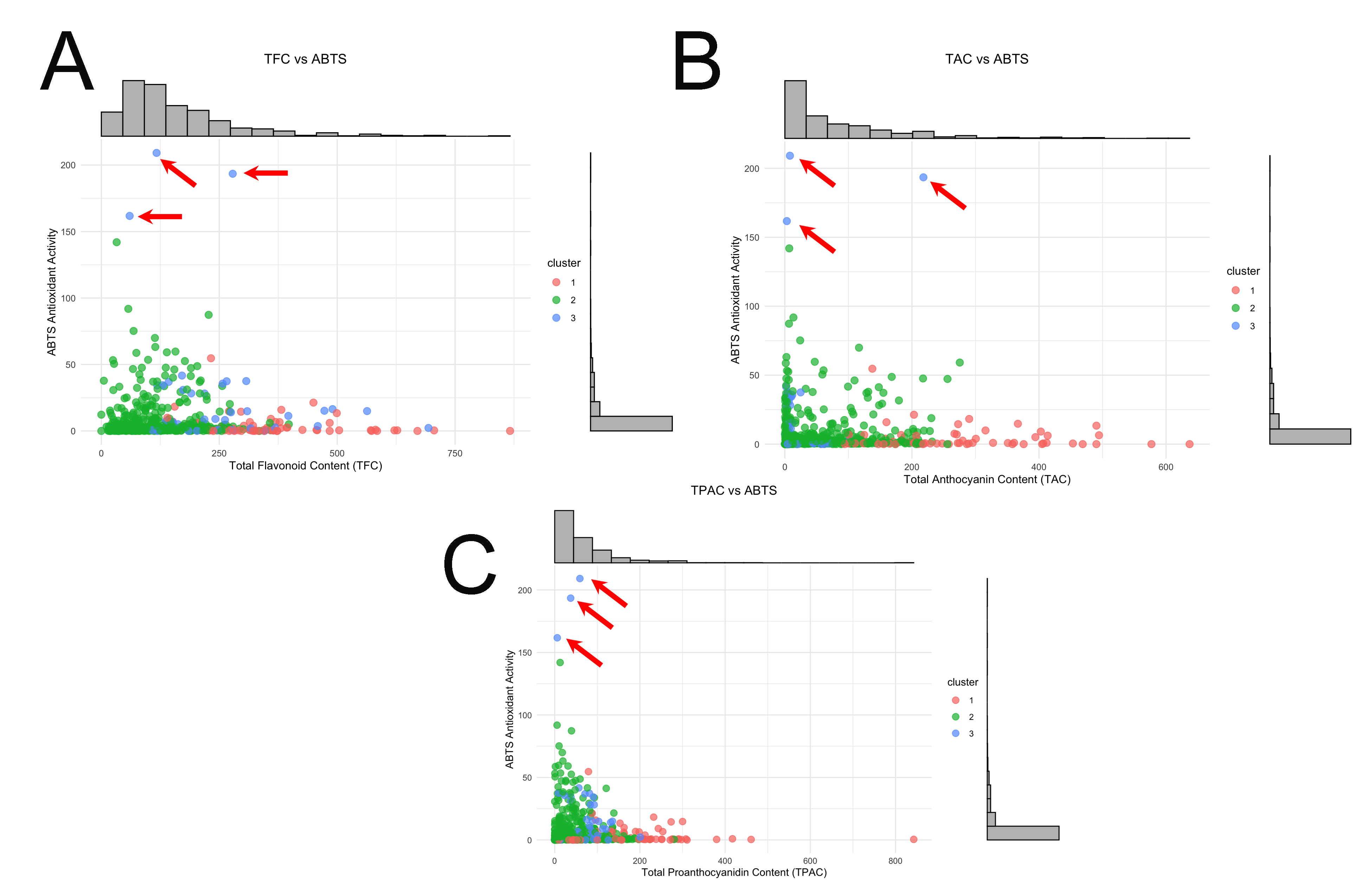


**Supplementary Figure 2.** Scatterplot and histogram of antioxidant components (A) total flavonoid content, (B) total anthocyanin content, and (C) total proanthocyanidin content


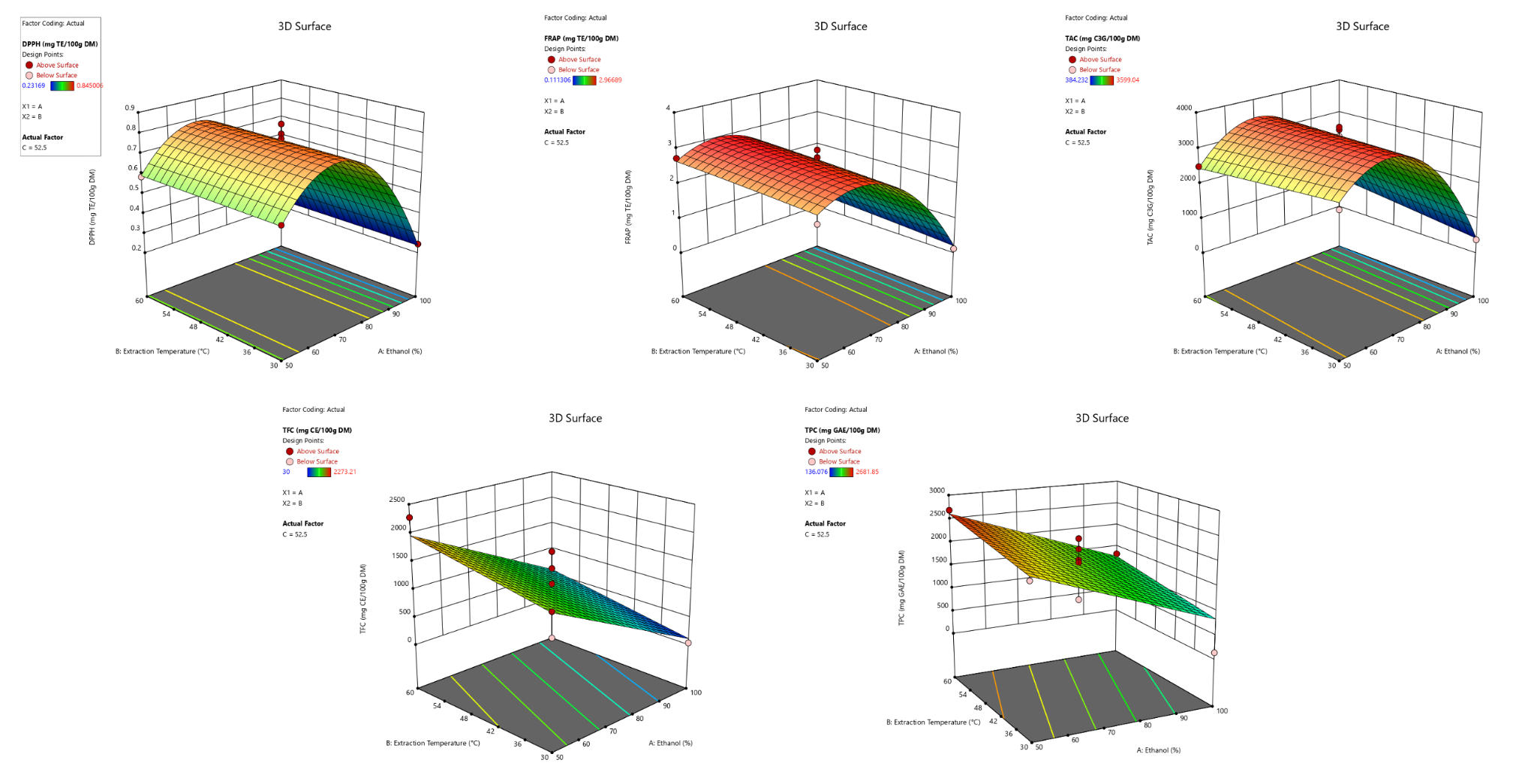
**Supplementary Figure 3**. Response surface methodology (RSM) for the optimization of rice bran extraction from the pigmented rice
