# Supplementary material for: Optimization of phenolic extraction method and in vitro bioaccessibility of microencapsulated pigmented rice bran extracts and their antioxidant and anticancer properties
Source: Food Hydrocoll Health. 2025 Jun;7:None. doi: 10.1016/j.fhfh.2025.100221 (PMC12167174; doi:10.1016/j.fhfh.2025.100221)
Supplement: Supplementary file 2 [file mmc2.docx]

**Supplementary Table 1. Solution scenarios resulting from response surface methodology - box behnken design optimized extraction parameters (51.244% Ethanol, 60°C, 1 Hour).**

| **Number** | **Ethanol, %** | **Extraction Temperature, ^o^C** | **Extraction Time, min** | **TPC, mg GAE /100g** | **TFC, mg CE /100g** | **TAC, mg C3G /100g** | **DPPH, mg TE/100g** | **FRAP, mg TE/100g** | **Desirability** |
| --- | --- | --- | --- | --- | --- | --- | --- | --- | --- |
| 1 | 51.244 | 60.000 | 60.000 | 2643.217 | 1910.130 | 2471.268 | 0.584 | 2.608 | 0.924 |
| 2 | 51.405 | 60.000 | 60.000 | 2638.931 | 1905.722 | 2487.401 | 0.588 | 2.616 | 0.924 |
| 3 | 51.045 | 60.000 | 60.000 | 2648.531 | 1915.596 | 2451.068 | 0.581 | 2.597 | 0.924 |
| 4 | 51.635 | 60.000 | 60.000 | 2632.790 | 1899.406 | 2510.273 | 0.592 | 2.628 | 0.924 |
| 5 | 50.777 | 60.000 | 60.000 | 2655.779 | 1923.051 | 2423.168 | 0.575 | 2.582 | 0.924 |
| 6 | 50.741 | 60.000 | 60.000 | 2656.652 | 1923.948 | 2419.786 | 0.574 | 2.581 | 0.924 |
| 7 | 51.845 | 60.000 | 60.000 | 2627.206 | 1893.665 | 2530.826 | 0.596 | 2.639 | 0.924 |
| 8 | 52.048 | 60.000 | 60.000 | 2621.794 | 1888.098 | 2550.518 | 0.600 | 2.649 | 0.924 |

**Supplementary Table 2. Corresponding codes of the factors used in research surface methodology for optimization of the extraction method.**

| **Variables** | **Symbol (code)** | **Levels** | | |
| --- | --- | --- | --- | --- |
|  |  | **-1** | **0** | **1** |
| % Ethanol | A | 50 | 75 | 100 |
| Extraction Temperature | B | 30 | 45 | 60 |
| Extraction Time | C | 45 | 52.5 | 60 |

**Supplementary Table 3.** **Fractionation parameters.**

| **Time (min)** | **% acetonitrile** | **% water** |
| --- | --- | --- |
| 0-2 | 5 | 95 |
| 2-12 | 70 | 30 |
| 12-16 | 100 | 0 |
| 16-22 | 100 | 0 |
| 22-24 | 5 | 95 |
| 24-34 | 5 | 0 |

**Supplementary Table 4. Metabolite analysis of the first two fractions.**

| Compound_class | Compound_name | mz_mean | RT_mean | G1B_T1 | G1B_T2 | G1B_T3 | G2B_T1 | G2B_T2 | G2B_T3 |
| --- | --- | --- | --- | --- | --- | --- | --- | --- | --- |
|  |  |  |  | G1B | G1B | G1B | G2B | G2B | G2B |
| Phenylpropanoids and polyketides | 3,4-Dihydroxyhydrocinnamic acid | 181.046999 | 5.30601588 | 1133406.4 | 1130810.4 | 1134150.4 | 4340237.5 | 4342792.5 | 4339386.5 |
| Phenylpropanoids and polyketides | 3,4-Dihydroxyphenylacetic acid | 167.033646 | 2.08907363 | 79563.625 | 76344.625 | 82415.625 | 205493.16 | 206938.16 | 203134.16 |
| Phenols | 4-Hydroxy-3-Methoxymandelic acid | 197.044369 | 2.91036266 | 531357.2 | 530282.2 | 532907.2 | 571890 | 575011 | 568654 |
| Phenols | 4-Hydroxymethyl-3-methoxyphenoxyacetic acid | 211.02376 | 4.39609563 | 211651.64 | 208950.64 | 214691.64 | 175285.92 | 178045.92 | 173731.92 |
| Glycosides | 4-Methoxyglucobrassicin | 477.059681 | 6.40859786 | 0 | 0 | 0 | 128939.76 | 129685.76 | 127920.76 |
| Phenolic acids | 4-O-pcoumaroylshikimic acid | 319.045075 | 6.40950877 | 4873798.5 | 4871508.5 | 4876046.5 | 32458812 | 32461420 | 32455639 |
| Phenylpropanoids and polyketides | Ampelopsin | 319.045109 | 6.11877594 | 0 | 0 | 0 | 0 | 0 | 0 |
| Phenylpropanoids and polyketides | Apigenin | 269.044887 | 10.5071103 | 0 | 0 | 0 | 0 | 0 | 0 |
| Phenylpropanoids and polyketides | Apigenin-7-O-glucoside | 431.139924 | 7.90456316 | 23922.602 | 23362.602 | 27151.602 | 34147.082 | 35366.082 | 31799.082 |
| Phenylpropanoids and polyketides | Astragalin | 447.085108 | 8.33133101 | 0 | 0 | 0 | 0 | 0 | 0 |
| Phenylpropanoids and polyketides | Caffeic acid | 179.033744 | 5.41715763 | 529329.4 | 527649.4 | 531430.4 | 188571.25 | 190310.25 | 186236.25 |
| Phenylpropanoids and polyketides | Caffeoyl spermidine derivative | 242.175275 | 11.1027881 | 84102848 | 84100999 | 84104853 | 97140008 | 97141117 | 97137720 |
| Phenylpropanoids and polyketides | caffeoylquinic acid dervitives | 515.094877 | 5.57298193 | 0 | 0 | 0 | 0 | 0 | 0 |
| Phenylpropanoids and polyketides | CafHex1_3.87_341.0879 | 341.107812 | 4.38319689 | 4129008.2 | 4126031.2 | 4132363.2 | 5089467 | 5092754 | 5088299 |
| Phenylpropanoids and polyketides | CafHex2_4.09_341.0879 | 341.107874 | 4.56409959 | 2447628 | 2446635 | 2448193 | 3761248.2 | 3762765.2 | 3759360.2 |
| Phenylpropanoids and polyketides | Catechin | 289.071149 | 4.99728085 | 441113.75 | 438611.75 | 442842.75 | 418856 | 419387 | 415644 |
| Phenylpropanoids and polyketides | chlorogenic acid | 353.086932 | 4.88929736 | 1003981.4 | 1000700.4 | 1005492.4 | 262664.97 | 264252.97 | 260440.97 |
| Phenolic acid | Chlorogenic acid derv. | 353.087004 | 5.15740436 | 235063.33 | 234153.33 | 236359.33 | 974040.94 | 974758.94 | 973206.94 |
| Phenylpropanoids and polyketides | Chrysoeriol 5-O-hexoside | 461.074966 | 5.64783812 | 0 | 0 | 0 | 623440.2 | 624613.2 | 620210.2 |
| Phenolic acid | cinnamic acid derivative | 265.147244 | 14.6767818 | 156444960 | 156442902 | 156447259 | 346751872 | 346755096 | 346750295 |
| Phenylpropanoids and polyketides | coumarin derivatives | 147.043833 | 7.38849924 | 858931.56 | 858187.56 | 860165.56 | 0 | 0 | 0 |
| Phenylpropanoids and polyketides | Coumaroylgalactarate_355.067_3.73 | 355.069498 | 3.74636699 | 170330.9 | 169773.9 | 173754.9 | 0 | 0 | 0 |
| Phenylpropanoids and polyketides | Cyanidin-3-O-glucoside | 465.102471 | 4.99736353 | 383647.28 | 382166.28 | 385186.28 | 89266.555 | 92549.555 | 87917.555 |
| Phenylpropanoids and polyketides | Cyanidin-3-O-sophoroside | 609.145225 | 4.75338076 | 23406.713 | 21452.713 | 26219.713 | 0 | 0 | 0 |
| Phenylpropanoids and polyketides | Delphinidin-3-O-galactoside | 463.099697 | 4.50826126 | 0 | 0 | 0 | 10688.153 | 11315.153 | 7664.153 |
| Phenylpropanoids and polyketides | delphinidin-3-O-rutinoside chloride | 609.145225 | 4.75338076 | 23406.713 | 21526.713 | 26764.713 | 0 | 0 | 0 |
| Phenylpropanoids and polyketides | Dihydromyricetin | 319.045011 | 5.77767507 | 0 | 0 | 0 | 0 | 0 | 0 |
| Phenylpropanoids and polyketides | FerHex2_4.65_355.1031 | 355.138882 | 5.22029844 | 0 | 0 | 0 | 0 | 0 | 0 |
| Phenylpropanoids and polyketides | FerHex3_5.17_355.1031 | 355.102717 | 5.76746556 | 673542.6 | 671599.6 | 674269.6 | 7453041 | 7455661 | 7449754 |
| Phenylpropanoids and polyketides | feruloyl-O-sinapoyl-O-caffeoylquinic acid | 735.156148 | 4.47291239 | 753957.25 | 750510.25 | 756424.25 | 157455.25 | 160574.25 | 156387.25 |
| Phenylpropanoids and polyketides | fragment ofC-hexosyl-luteolin O-hexoside | 299.018991 | 9.93841442 | 29161956 | 29158552 | 29164795 | 368106400 | 368109222 | 368104296 |
| phenolic acid | gallic acid | 169.012968 | 2.64050297 | 105372.055 | 104173.055 | 106773.055 | 0 | 0 | 0 |
| Phenylpropanoids and polyketides | H5FeruGlc1_3.92_371.0983 | 371.097443 | 4.36781439 | 0 | 0 | 0 | 445957.16 | 447698.16 | 445121.16 |
| Phenylpropanoids and polyketides | Hesperetin-7-O-glucoside | 463.08737 | 8.20462532 | 0 | 0 | 0 | 0 | 0 | 0 |
| Phenylpropanoids and polyketides | hesperidine | 609.181621 | 7.89326704 | 0 | 0 | 0 | 779109.94 | 782522.94 | 777997.94 |
| Phenylpropanoids and polyketides | Homoeriodictyol | 301.071031 | 9.51742617 | 0 | 0 | 0 | 0 | 0 | 0 |
| Phenylpropanoids and polyketides | Hydroxygallic acid derivatives | 187.14279 | 8.02836518 | 1495385.6 | 1494465.6 | 1497838.6 | 1289478.2 | 1292501.2 | 1286371.2 |
| Phenylpropanoids and polyketides | Is3G_7.29_477.103 | 477.102738 | 7.80088074 | 231154.55 | 227953.55 | 232894.55 | 2370649 | 2373581 | 2368891 |
| Phenylpropanoids and polyketides | Is3G2-A_6.79_609.1467 | 609.17934 | 7.16156993 | 0 | 0 | 0 | 0 | 0 | 0 |
| Phenylpropanoids and polyketides | Is3G6-R_7.02_623.1614 | 623.139965 | 7.61096568 | 0 | 0 | 0 | 0 | 0 | 0 |
| Phenylpropanoids and polyketides | Is3G6-R7G_5.22_785.2141 | 785.158834 | 5.75389079 | 0 | 0 | 0 | 0 | 0 | 0 |
| Phenylpropanoids and polyketides | isorhamentin | 315.050302 | 10.9724313 | 216382.31 | 214777.31 | 217178.31 | 0 | 0 | 0 |
| Phenylpropanoids and polyketides | Isorhamnetin-3-O-glucoside | 477.102738 | 7.80088074 | 231154.55 | 229587.55 | 232131.55 | 2370649 | 2373544 | 2367680 |
| Phenylpropanoids and polyketides | Isorhamnetin-3-O-rutinoside | 623.158865 | 7.41952357 | 0 | 0 | 0 | 0 | 0 | 0 |
| Phenylpropanoids and polyketides | Isorhamnetin-3-O-rutinoside | 623.139965 | 7.61096568 | 0 | 0 | 0 | 0 | 0 | 0 |
| Phenylpropanoids and polyketides | isorhamnetin-o-glucoside | 477.102738 | 7.80088074 | 231154.55 | 229131.55 | 233202.55 | 2370649 | 2373890 | 2368240 |
| Phenylpropanoids and polyketides | K3G_7.13_447.0926 | 447.053513 | 7.69766989 | 0 | 0 | 0 | 0 | 0 | 0 |
| Phenylpropanoids and polyketides | K3G7G_6.14_609.1462 | 609.195775 | 6.77115581 | 0 | 0 | 0 | 0 | 0 | 0 |
| Phenylpropanoids and polyketides | Kaempferol | 287.018658 | 9.25863034 | 0 | 0 | 0 | 0 | 0 | 0 |
| Phenylpropanoids and polyketides | Kaempferol 3-O-glucoside 7-O-rhamnoside | 593.150364 | 6.64338208 | 0 | 0 | 0 | 1894900.5 | 1897361.5 | 1892458.5 |
| Phenylpropanoids and polyketides | Kaempferol 3-O-rhamnoside-7-O-glucoside | 593.171799 | 5.78780606 | 0 | 0 | 0 | 0 | 0 | 0 |
| Phenylpropanoids and polyketides | Kaempferol-3-O-?-rutinoside | 593.205262 | 7.54231587 | 0 | 0 | 0 | 0 | 0 | 0 |
| Phenylpropanoids and polyketides | Kaempferol-3-O-Glucoside | 447.053513 | 7.69766989 | 0 | 0 | 0 | 0 | 0 | 0 |
| Phenylpropanoids and polyketides | Kaempferol-3-O-Glucoside | 447.053513 | 7.69766989 | 0 | 0 | 0 | 0 | 0 | 0 |
| Phenylpropanoids and polyketides | kaempferol-3-rhamnosyhexose | 593.186264 | 9.41033348 | 0 | 0 | 0 | 0 | 0 | 0 |
| Phenylpropanoids and polyketides | Kaempferol-7-O-Glucoside | 447.053513 | 7.69766989 | 0 | 0 | 0 | 0 | 0 | 0 |
| Phenylpropanoids and polyketides | Kaempferol-7-O-Glucoside | 447.053513 | 7.69766989 | 0 | 0 | 0 | 0 | 0 | 0 |
| Phenylpropanoids and polyketides | kaempferol-glycosides derv. | 593.186264 | 9.41033348 | 0 | 0 | 0 | 0 | 0 | 0 |
| Phenylpropanoids and polyketides | kaempferol-glycosides dervi | 755.131935 | 7.67940411 | 0 | 0 | 0 | 0 | 0 | 0 |
| Phenylpropanoids and polyketides | kaempferol-glycosides dervi | 755.131935 | 7.67940411 | 0 | 0 | 0 | 0 | 0 | 0 |
| Phenylpropanoids and polyketides | KGRAFer_7.93_901.2408 | 901.297578 | 8.42209493 | 0 | 0 | 0 | 0 | 0 | 0 |
| Phenylpropanoids and polyketides | Luteolin 6-C glucoside | 449.274568 | 10.8650551 | 544.3796 | -511.6204 | 1387.3796 | 0 | 0 | 0 |
| Phenylpropanoids and polyketides | Morin | 301.081512 | 8.78877692 | 0 | 0 | 0 | 0 | 0 | 0 |
| Phenylpropanoids and polyketides | Myricetin | 317.054629 | 8.39225873 | 0 | 0 | 0 | 0 | 0 | 0 |
| Phenylpropanoids and polyketides | Naringenin chalcone | 271.060562 | 10.3978861 | 0 | 0 | 0 | 0 | 0 | 0 |
| Phenylpropanoids and polyketides | Naringin | 579.17069 | 7.91211264 | 0 | 0 | 0 | 0 | 0 | 0 |
| Phenylpropanoids and polyketides | NG1_7.39_433.1136 | 433.112916 | 7.86818572 | 0 | 0 | 0 | 0 | 0 | 0 |
| Phenylpropanoids and polyketides | P35GG_6.64_597.18 | 597.181506 | 7.23250482 | 0 | 0 | 0 | 0 | 0 | 0 |
| Phenylpropanoids and polyketides | peak118_Phaseoloidin_329.0875_3.74 | 329.08707 | 3.62928104 | 1942345.2 | 1938891.2 | 1943193.2 | 6238753.5 | 6242201.5 | 6237622.5 |
| Phenylpropanoids and polyketides | peak120_Licoagroside B_431.1192_3.75 | 431.118638 | 3.78433284 | 79726.85 | 77887.85 | 81477.85 | 0 | 0 | 0 |
| Phenylpropanoids and polyketides | peak131_Vanilloside_313.0927659_3.96 | 313.092066 | 3.98115873 | 91553.95 | 88437.95 | 94346.95 | 194470.98 | 197921.98 | 193163.98 |
| Phenylpropanoids and polyketides | peak157_Coumaroylgalactarate_355.067_4.26 | 355.102582 | 4.28907692 | 0 | 0 | 0 | 0 | 0 | 0 |
| Phenylpropanoids and polyketides | peak173_Coumaroylgalactarate_355.067_4.52 | 355.066217 | 4.56168596 | 133104.1 | 131409.1 | 134507.1 | 317733.4 | 320880.4 | 315159.4 |
| Phenylpropanoids and polyketides | peak185_Feruloylgalactarate_385.0777_4.63 | 385.076741 | 4.65001032 | 0 | 0 | 0 | 0 | 0 | 0 |
| Phenylpropanoids and polyketides | peak196_1-O-Feruloyl-glucose_355.1034_4.76 | 355.102482 | 4.77409233 | 0 | 0 | 0 | 0 | 0 | 0 |
| Phenylpropanoids and polyketides | peak197_Dihydrophaseic acid glucoside_443.1922_4.77 | 443.191254 | 4.77388608 | 0 | 0 | 0 | 323694.06 | 326905.06 | 320920.06 |
| Phenylpropanoids and polyketides | peak203_Glucocaffeic acid_341.0876_4.87 | 341.086746 | 4.88097752 | 508201.06 | 507163.06 | 509354.06 | 195848.44 | 197182.44 | 193574.44 |
| Phenylpropanoids and polyketides | peak205_p-coumaroyl tartaric acid_295.0457_4.88 | 295.045075 | 4.92405396 | 784051.6 | 780939.6 | 787269.6 | 0 | 0 | 0 |
| Phenylpropanoids and polyketides | peak217_Sinapoylgalactarate_385.0777_4.96 | 385.076712 | 4.98475331 | 0 | 0 | 0 | 205800.9 | 208352.9 | 204429.9 |
| Phenylpropanoids and polyketides | peak223_Epicatechin_289.0716986_4.98 | 289.071149 | 4.99728085 | 441113.75 | 438382.75 | 442470.75 | 418856 | 419419 | 417397 |
| Phenylpropanoids and polyketides | peak229_p-Coumaroyl-glucose_325.0928455_5.06 | 325.092003 | 5.07326054 | 316637.7 | 314510.7 | 318091.7 | 1935794.2 | 1937510.2 | 1933408.2 |
| Phenylpropanoids and polyketides | peak256_Licoagroside B_431.1191_5.32 | 431.139313 | 5.42022078 | 124472.055 | 121504.055 | 126178.055 | 62212.664 | 63099.664 | 60972.664 |
| Phenylpropanoids and polyketides | peak257_Fertaric acid_325.0563_5.32 | 325.092104 | 5.34276532 | 0 | 0 | 0 | 1329625.2 | 1332952.2 | 1328112.2 |
| Phenylpropanoids and polyketides | peak270_Feruloylglucose_355.1033126_5.45 | 355.102481 | 5.46695846 | 568787 | 566406 | 571727 | 4299461.5 | 4300933.5 | 4297003.5 |
| Phenylpropanoids and polyketides | peak272_hydroxyjasmonic acid glucoside_387.166_5.47 | 387.113501 | 5.43290896 | 19280000 | 19279480 | 19282247 | 11103584 | 11105066 | 11101759 |
| Phenylpropanoids and polyketides | peak297__449.10889_5.76 | 449.105435 | 5.8632348 | 0 | 0 | 0 | 13926.117 | 14547.117 | 12665.117 |
| Phenylpropanoids and polyketides | peak301_Feruloyl di-glucoside_517.1565_5.78 | 517.155047 | 5.7802043 | 2710442.5 | 2708110.5 | 2713181.5 | 54001268 | 54004470 | 53998650 |
| Phenylpropanoids and polyketides | peak314_Caffeoylshikimic acid_335.0771_5.91 | 335.133989 | 5.94141458 | 10624.857 | 7983.857 | 11650.857 | 0 | 0 | 0 |
| Phenylpropanoids and polyketides | peak364_Quercetin-p-coumarylglucoside putative_609.1464941_6.56 | 609.145495 | 6.45105755 | 379108.62 | 376952.62 | 380525.62 | 8085715.5 | 8087613.5 | 8083341.5 |
| Phenylpropanoids and polyketides | peak373_Kaempferol-rha-glc-xyl_725.1943_6.65 | 725.15493 | 6.65150125 | 0 | 0 | 0 | 0 | 0 | 0 |
| Phenylpropanoids and polyketides | peak379_p-coumaroylmalate_279.0509_6.75 | 279.050319 | 6.77639313 | 21694646 | 21691923 | 21697422 | 670322.25 | 673223.25 | 668587.25 |
| Phenylpropanoids and polyketides | peak383_4-Coumaroylshikimate_319.082_6.79 | 319.138877 | 6.80763831 | 0 | 0 | 0 | 119624.42 | 120492.42 | 118832.42 |
| Phenylpropanoids and polyketides | peak384_Quercetin-rha-glc_609.1468_6.82 | 609.145403 | 6.82142105 | 0 | 0 | 0 | 2220298 | 2222165 | 2218296 |
| Phenylpropanoids and polyketides | peak389_p-coumaroylmalate_279.0509_6.93 | 279.050345 | 6.95814309 | 20233284 | 20232321 | 20236596 | 0 | 0 | 0 |
| Phenylpropanoids and polyketides | peak401_Quercetin-glc_463.0884_7.05 | 463.08035 | 6.98807432 | 101580.11 | 100841.11 | 104673.11 | 1146482.8 | 1148757.8 | 1145817.8 |
| Phenylpropanoids and polyketides | peak403_Laricitrin-glc like_493.0988_7.11 | 493.097953 | 7.19824103 | 0 | 0 | 0 | 0 | 0 | 0 |
| Phenylpropanoids and polyketides | peak406_Phaselic acid+methyl_309.0615_7.15 | 309.082083 | 7.23197141 | 928648.8 | 926008.8 | 931458.8 | 54925.71 | 56094.71 | 54182.71 |
| Phenylpropanoids and polyketides | peak414_Genistein-glc_431.0989_7.26 | 431.133423 | 7.24447914 | 41603.42 | 39534.42 | 42636.42 | 80308.45 | 82616.45 | 77515.45 |
| Phenylpropanoids and polyketides | peak417_4-Coumaroylshikimate_319.0821_7.31 | 319.081467 | 7.26541346 | 0 | 0 | 0 | 214538.8 | 217706.8 | 211198.8 |
| Glycosides | peak439_Isorhamnetin-rha-glc_623.1622_7.53 | 623.139965 | 7.61096568 | 0 | 0 | 0 | 0 | 0 | 0 |
| Glycosides | peak453_Kaempferol-glc_447.0932_7.64 | 447.053513 | 7.69766989 | 0 | 0 | 0 | 0 | 0 | 0 |
| Glycosides | peak453_Kaempferol-glc_447.0932_7.64 | 447.053513 | 7.69766989 | 0 | 0 | 0 | 0 | 0 | 0 |
| Glycosides | peak467_Kaempferol glu_447.093162_7.77 | 447.053513 | 7.69766989 | 0 | 0 | 0 | 0 | 0 | 0 |
| Glycosides | peak468_Methyl-Quercetin-glc_477.1038_7.8 | 477.102738 | 7.80088074 | 231154.55 | 228938.55 | 233081.55 | 2370649 | 2373770 | 2369462 |
| Glycosides | peak539_p-coumaroyl-sinapoyl di-glucoside_693.2045_8.56 | 693.238685 | 8.80288368 | 0 | 0 | 0 | 0 | 0 | 0 |
| Glycosides | peak638_p-coumaroyl-sinapoyl glucoside_531.1509_9.78 | 531.18628 | 9.67260512 | 0 | 0 | 0 | 0 | 0 | 0 |
| Glycosides | peak77_Galloylglucose_331.0669_3.14 | 331.066369 | 3.23035174 | 54956.594 | 54360.594 | 57718.594 | 0 | 0 | 0 |
| Phenylpropanoids and polyketides | Phaseoloidin_329.0875_3.74 | 329.086875 | 3.76387602 | 18939442 | 18937743 | 18941919 | 4434576.5 | 4437569.5 | 4431554.5 |
| phenolic acid | Protocatechuic acid | 153.052508 | 3.78369841 | 702073.44 | 698717.44 | 704242.44 | 3092303.5 | 3092916.5 | 3091627.5 |
| Glycosides | Q3G_6.56_463.0878 | 463.123858 | 7.09579536 | 0 | 0 | 0 | 425533.1 | 428699.1 | 422634.1 |
| Glycosides | Q3G2-A_6.19_595.1315 | 595.129365 | 6.64646569 | 0 | 0 | 0 | 0 | 0 | 0 |
| Glycosides | Q3G6-R_6.33_609.1458 | 609.145403 | 6.82142105 | 0 | 0 | 0 | 2220298 | 2222063 | 2217438 |
| Glycosides | Q3G7G_5.68_625.1412 | 625.176227 | 6.19800469 | 0 | 0 | 0 | 0 | 0 | 0 |
| Phenylpropanoids and polyketides | Quercelin-3-unk. | 463.08035 | 6.98807432 | 101580.11 | 100453.11 | 104739.11 | 1146482.8 | 1149075.8 | 1145671.8 |
| Phenylpropanoids and polyketides | Quercetin | 301.034488 | 7.0418385 | 118431.03 | 116098.03 | 119615.03 | 1569.4327 | 3125.4327 | -1069.5673 |
| Glycosides | Quercetin-3-?-D-Glucoside | 463.08035 | 6.98807432 | 101580.11 | 98559.11 | 104798.11 | 1146482.8 | 1147419.8 | 1144665.8 |
| Glycosides | Quercetin-3-beta-D-glucoside | 463.123858 | 7.09579536 | 0 | 0 | 0 | 425533.1 | 427938.1 | 424784.1 |
| Glycosides | Quercetin-3-D-galactoside | 463.08035 | 6.98807432 | 101580.11 | 100617.11 | 104065.11 | 1146482.8 | 1149144.8 | 1144679.8 |
| Glycosides | quercetin-7-O-hexoside | 465.083325 | 5.33823928 | 0 | 0 | 0 | 0 | 0 | 0 |
| Glycosides | Quercetin-apiosyl-galactose | 595.129365 | 6.64646569 | 0 | 0 | 0 | 0 | 0 | 0 |
| Glycosides | Quercetine -glucosides derv. | 625.17668 | 6.9437791 | 0 | 0 | 0 | 0 | 0 | 0 |
| Phenylpropanoids and polyketides | Quercitrin | 447.053513 | 7.69766989 | 0 | 0 | 0 | 0 | 0 | 0 |
| Phenylpropanoids and polyketides | Quercitrin | 447.053513 | 7.69766989 | 0 | 0 | 0 | 0 | 0 | 0 |
| Phenylpropanoids and polyketides | Rutin | 609.145403 | 6.82142105 | 0 | 0 | 0 | 2220298 | 2223496 | 2218572 |
| Phenylpropanoids and polyketides | Rutin | 609.145403 | 6.82142105 | 0 | 0 | 0 | 2220298 | 2220971 | 2219768 |
| Glycosides | Salicin | 285.060926 | 4.36543663 | 89018.734 | 86449.734 | 92477.734 | 0 | 0 | 0 |
| Phenylpropanoids and polyketides | Secoisolariciresinol | 361.201039 | 12.4328636 | 0 | 0 | 0 | 0 | 0 | 0 |
| Glycosides | Sinapoylglucose | 385.113151 | 5.6038441 | 1653217.6 | 1650075.6 | 1656064.6 | 20812730 | 20813726 | 20811160 |
| Glycosides | SinGlc_5.24_385.1133 | 385.113178 | 5.89819111 | 0 | 0 | 0 | 672802.94 | 673777.94 | 669488.94 |
| Glycosides | SinGlciso2_5.47_385.1133 | 385.092803 | 6.02332622 | 0 | 0 | 0 | 0 | 0 | 0 |
| Glycosides | trans-zeatin-O-glucoside | 380.155299 | 3.86441777 | 18273114 | 18271634 | 18274074 | 2013877.8 | 2016625.8 | 2011692.8 |
| Phenylpropanoids and polyketides | Tricetin | 301.070857 | 10.651752 | 133921.75 | 131223.75 | 136492.75 | 0 | 0 | 0 |
| Phenylpropanoids and polyketides | tricin | 329.244156 | 11.5355308 | 0 | 0 | 0 | 0 | 0 | 0 |
| phenolic acid | vanillic acid | 167.033638 | 7.25285866 | 84569.49 | 81290.49 | 87118.49 | 46524.434 | 48281.434 | 45100.434 |
| Phenylpropanoids and polyketides | Vitexin-2"-O-rhamnoside | 577.116574 | 6.73757738 | 0 | 0 | 0 | 0 | 0 | 0 |

**Supplementary Table 5.** The mean total phenolic content (TPC) and antioxidant ABTS of 542 dehusked samples. Zero values mean below the detection limit.

| No. | Code | mean TPC (mg GAE/100 g) | mean ABTS (mg trolox equiv/g extract) |
| --- | --- | --- | --- |
| 1 | MBCR1 | 1455.717655 | 14.75016096 |
| 2 | MBCR4 | 100.1675421 | 15.04979711 |
| 3 | MBCR5 | 274.4848485 | 3.783073714 |
| 4 | MBCR6 | 290.6666666 | 6.724812142 |
| 5 | MBCR7 | 157.939394 | 14.59182019 |
| 6 | MBCR8 | 162.060606 | 14.59711919 |
| 7 | MBCR9 | 177.5757576 | 25.1890041 |
| 8 | MBCR10 | 256 | 5.073707975 |
| 9 | MBCR11 | 378.4848485 | 21.57091838 |
| 10 | MBCR13 | 330.7272727 | 17.49144595 |
| 11 | MBCR14 | 370.6955413 | 9.79229036 |
| 12 | MBCR15 | 268.2424243 | 7.273078492 |
| 13 | MBCR16 | 384.1358567 | 18.84234004 |
| 14 | MBCR17 | 107.426532 | 17.163652 |
| 15 | MBCR19 | 171.5151515 | 7.889110949 |
| 16 | MBCR21 | 243.2727273 | 0.78917191 |
| 17 | MBCR23 | 347.7703704 | 0.37815979 |
| 18 | MBCR24 | 381.4545454 | 9.269969978 |
| 19 | MBCR25 | 341.2727276 | 41.28092777 |
| 20 | MBCR26 | 257.9901647 | 8.617100549 |
| 21 | MBCR29 | 172.4848485 | 7.719619245 |
| 22 | MBCR30 | 70.67469136 | 0.750433066 |
| 23 | MBCR31 | 227.5757576 | 8.478027926 |
| 24 | MBCR32 | 228.6060606 | 22.59090226 |
| 25 | MBCR33 | 274.7272727 | 13.50851327 |
| 26 | MBCR34 | 604.3928389 | 6.144403297 |
| 27 | MBCR35 | 389.4974644 | 9.494463703 |
| 28 | MBCR36 | 781.122943 | 18.26058664 |
| 29 | MBCR37 | 392.5558388 | 7.53523612 |
| 30 | MBCR39 | 515.719798 | 21.53375863 |
| 31 | MBCR40 | 224.7272728 | 33.67962398 |
| 32 | MBCR41 | 360.9359759 | 9.205806902 |
| 33 | MBCR42 | 134.9174411 | 9.442258077 |
| 34 | MBCR43 | 261.7587113 | 11.0179544 |
| 35 | MBCR44 | 476.8724532 | 10.51902586 |
| 36 | MBCR45 | 637.8486419 | 5.066342013 |
| 37 | MBCR46 | 389.2283262 | 7.013383509 |
| 38 | MBCR47 | 701.8502577 | 6.452370702 |
| 39 | MBCR48 | 259.6969697 | 1.580968237 |
| 40 | MBCR49 | 813.1515152 | 9.09768717 |
| 41 | MBCR50 | 219.3532516 | 9.878606341 |
| 42 | MBCR51 | 560.3636364 | 3.303102968 |
| 43 | MBCR52 | 577.3939394 | 1.754844748 |
| 44 | MBCR53 | 828.8662626 | 6.69007004 |
| 45 | MBCR54 | 238.8293206 | 11.7571803 |
| 46 | MBCR55 | 430.1212121 | 29.3446523 |
| 47 | MBCR56 | 293.8512633 | 21.77534203 |
| 48 | MBCR57 | 606.5847349 | 21.32146234 |
| 49 | MBCR58 | 442.3409609 | 1.994715482 |
| 50 | MBCR59 | 298.4261413 | 1.948054406 |
| 51 | MBCR60 | 392.6583217 | 14.5294076 |
| 52 | MBCR61 | 59.27160494 | 37.79563287 |
| 53 | MBCR62 | 257.3378307 | 0.932523077 |
| 54 | MBCR63 | 117.341358 | 2.555280221 |
| 55 | MBCR65 | 86.29567896 | 1.992961725 |
| 56 | MBCR67 | 279.8015873 | 1.64289962 |
| 57 | MBCR69 | 149.957673 | 1.520222754 |
| 58 | MBCR70 | 257.2786597 | 4.716925105 |
| 59 | MBCR71 | 549.3548315 | 2.551085 |
| 60 | MBCR72 | 183.7022217 | 1.415481951 |
| 61 | MBCR74 | 323.0563193 | 3.49062664 |
| 62 | MBCR75 | 262.2659202 | 3.358229003 |
| 63 | MBCR76 | 358.7020141 | 2.360739355 |
| 64 | MBCR77 | 142.6445053 | 5.866477739 |
| 65 | MBCR79 | 467.6337697 | 2.150126607 |
| 66 | MBCR80 | 143.9753086 | 3.270466596 |
| 67 | MBCR81 | 238.2518518 | 0 |
| 68 | MBCR82 | 163.292769 | 4.110961726 |
| 69 | MBCR83 | 326.9037037 | 0.712742879 |
| 70 | MBCR84 | 459.99148 | 1.877592656 |
| 71 | MBCR85 | 636.8322912 | 3.940071264 |
| 72 | MBCR87 | 133.2345679 | 4.607711174 |
| 73 | MBCR88 | 328.4296179 | 5.021310171 |
| 74 | MBCR89 | 285.2 | 1.740369511 |
| 75 | MBCR90 | 244.670055 | 4.597062892 |
| 76 | MBCR91 | 161.2645502 | 5.558711724 |
| 77 | MBCR92 | 278.7160494 | 4.885656924 |
| 78 | MBCR93 | 142.6006173 | 1.181770207 |
| 79 | MBCR94 | 279.4705646 | 2.680530217 |
| 80 | MBCR95 | 592.8795427 | 15.89345479 |
| 81 | MBCR98 | 245.4268078 | 3.05607148 |
| 82 | MBCR100 | 79.76728395 | 3.154862971 |
| 83 | MBCR101 | 607.2054881 | 2.412945914 |
| 84 | MBCR105 | 127.0229276 | 4.139538033 |
| 85 | MBCR106 | 411.2842612 | 2.511701845 |
| 86 | MBCR108 | 678.7485018 | 3.379920285 |
| 87 | MBCR109 | 168.2796296 | 0.975409974 |
| 88 | MBCR110 | 107.1407407 | 0.314951609 |
| 89 | MBCR111 | 312.8537037 | 0.531823781 |
| 90 | MBCR113 | 185.0917108 | 2.460129073 |
| 91 | MBCR114 | 132.313933 | 3.024207938 |
| 92 | MBCR115 | 491.305485 | 2.431873245 |
| 93 | MBCR116 | 151.7117284 | 1.176665467 |
| 94 | MBCR117 | 247.2619048 | 4.620050216 |
| 95 | MBCR118 | 214.0776014 | 5.633273989 |
| 96 | MBCR120 | 198.8492063 | 6.583955645 |
| 97 | MBCR121 | 76.52670111 | 1.798686295 |
| 98 | MBCR122 | 96.25185185 | 0.649244801 |
| 99 | MBCR123 | 345.8007779 | 2.015927639 |
| 100 | MBCR124 | 350.2768959 | 2.460156861 |
| 101 | MBCR125 | 305.5149912 | 2.21921043 |
| 102 | MBCR126 | 143.8972099 | 0.361281702 |
| 103 | MBCR128 | 142.2015943 | 2.335668071 |
| 104 | MBCR129 | 74.18694885 | 3.6092614 |
| 105 | MBCR130 | 40.7345679 | 3.106294452 |
| 106 | MBCR131 | 266.8633157 | 2.120506502 |
| 107 | MBCR132 | 264.6108729 | 2.358584077 |
| 108 | MBCR133 | 144.1116402 | 0.695220816 |
| 109 | MBCR134 | 80.11869489 | 0.656966312 |
| 110 | MBCR135 | 284.8447972 | 1.797442939 |
| 111 | MBCR136 | 241.2998236 | 0.668933779 |
| 112 | MBCR137 | 75.73518519 | 0.99193787 |
| 113 | MBCR138 | 187.9365079 | 3.256793062 |
| 114 | MBCR139 | 236.3819821 | 1.220560453 |
| 115 | MBCR142 | 724.264352 | 3.019660313 |
| 116 | MBCR143 | 133.0017637 | 1.820645958 |
| 117 | MBCR146 | 365.5842403 | 7.192978997 |
| 118 | MBCR147 | 269.7179552 | 4.871041427 |
| 119 | MBCR148 | 27.32589914 | 3.172516005 |
| 120 | MBCR149 | 263.7587301 | 1.146138264 |
| 121 | MBCR150 | 493.2031746 | 2.880120866 |
| 122 | MBCR151 | 77.3147463 | 2.167637322 |
| 123 | MBCR153 | 306.297231 | 1.669157217 |
| 124 | MBCR154 | 74.35246914 | 0.806385221 |
| 125 | MBCR155 | 214.0888889 | 0.768546863 |
| 126 | MBCR156 | 62.66137567 | 5.961307973 |
| 127 | MBCR157 | 118.5211949 | 1.716996657 |
| 128 | MBCR158 | 78.38236331 | 0.432910273 |
| 129 | MBCR159 | 229.9544974 | 0.616881253 |
| 130 | MBCR161 | 117.3527337 | 1.088229814 |
| 131 | MBCR164 | 95.99603175 | 0.271606023 |
| 132 | MBCR165 | 111.0917108 | 3.084428952 |
| 133 | MBCR166 | 93.38888889 | 2.571011978 |
| 134 | MBCR167 | 304.0515873 | 0.532334189 |
| 135 | MBCR168 | 61.10197561 | 2.088780195 |
| 136 | MBCR169 | 94.2707231 | 2.93826546 |
| 137 | MBCR170 | 63.87222222 | 2.612300293 |
| 138 | MBCR171 | 128.5851852 | 0.206850499 |
| 139 | MBCR172 | 391.7089948 | 0.533245896 |
| 140 | MBCR173 | 228.6476191 | 1.755290016 |
| 141 | MBCR176 | 106.2592593 | 0.478379568 |
| 142 | MBCR177 | 84.72619048 | 0.597400609 |
| 143 | MBCR179 | 250.2010582 | 0.47347825 |
| 144 | MBCR180 | 238.4991182 | 0.631898353 |
| 145 | MBCR181 | 273.3481481 | 3.071373629 |
| 146 | MBCR182 | 169.3809524 | 1.480469404 |
| 147 | MBCR183 | 242.0705468 | 1.009163178 |
| 148 | MBCR184 | 81.36948854 | 4.840961876 |
| 149 | MBCR186 | 283.1552028 | 0.500649969 |
| 150 | MBCR187 | 508.952381 | 0.724362954 |
| 151 | MBCR189 | 187.4339506 | 2.134398551 |
| 152 | MBCR190 | 106.7425926 | 0.213975673 |
| 153 | MBCR192 | 85.60061728 | 2.903582842 |
| 154 | MBCR194 | 254.3783069 | 0.056151568 |
| 155 | MBCR195 | 179.3059965 | 0.700074637 |
| 156 | MBCR196 | 102.4012346 | 0.885638864 |
| 157 | MBCR197 | 59.96296296 | 4.918961342 |
| 158 | MBCR199 | 82.26895943 | 0.284154217 |
| 159 | MBCR200 | 184.5396825 | 0.492923636 |
| 160 | MBCR201 | 116.4543651 | 0.331995389 |
| 161 | MBCR203 | 125.9285714 | 1.364932058 |
| 162 | MBCR204 | 182.6261023 | 0.553990293 |
| 163 | MBCR205 | 310.7610229 | 0.48192088 |
| 164 | MBCR206 | 380.6728395 | 0.56467046 |
| 165 | MBCR207 | 115.8712522 | 0.30956886 |
| 166 | MBCR208 | 245.3686067 | 0.46793432 |
| 167 | MBCR209 | 54.07671957 | 6.426472858 |
| 168 | MBCR210 | 225.0330262 | 0.721299116 |
| 169 | MBCR211 | 164.0426124 | 1.577572022 |
| 170 | MBCR212 | 181.8907407 | 0.519631818 |
| 171 | MBCR213 | 38.24876543 | 3.505017753 |
| 172 | MBCR214 | 108.5384127 | 4.713438913 |
| 173 | MBCR215 | 190.1574074 | 2.145200822 |
| 174 | MBCR216 | 249.733686 | 0.418754281 |
| 175 | MBCR217 | 145.9385997 | 1.378561103 |
| 176 | MBCR218 | 83.81746032 | 2.669975644 |
| 177 | MBCR219 | 91.62883598 | 2.631575336 |
| 178 | MBCR220 | 180.8104939 | 0.490139143 |
| 179 | MBCR221 | 54.26728395 | 1.909407338 |
| 180 | MBCR222 | 32.80432098 | 5.822914705 |
| 181 | MBCR223 | 61.19320988 | 4.304173409 |
| 182 | MBCR224 | 39.69320988 | 3.269503493 |
| 183 | MBCR225 | 513.8804233 | 6.649609503 |
| 184 | MBCR226 | 30.23024691 | 7.765820811 |
| 185 | MBCR227 | 62.31049382 | 2.229132914 |
| 186 | MBCR228 | 69.42344073 | 9.407813489 |
| 187 | MBCR229 | 208.9523809 | 1.867702912 |
| 188 | MBCR230 | 209.6582011 | 3.73950039 |
| 189 | MBCR231 | 80.19320988 | 6.99755634 |
| 190 | MBCR232 | 390.3724867 | 1.470389809 |
| 191 | MBCR233 | 132.6267067 | 2.692671817 |
| 192 | MBCR234 | 136.197619 | 1.778710462 |
| 193 | MBCR235 | 507.7071429 | 1.013803041 |
| 194 | MBCR237 | 136.5761561 | 2.732742406 |
| 195 | MBCR238 | 428.0634215 | 2.766733396 |
| 196 | MBCR239 | 222.3571429 | 1.976592883 |
| 197 | MBCR240 | 187.2145503 | 2.047782803 |
| 198 | MBCR241 | 114.010582 | 2.018300189 |
| 199 | MBCR242 | 465.1428572 | 3.896914541 |
| 200 | MBCR243 | 51.3606702 | 3.399161222 |
| 201 | MBCR244 | 412.1111111 | 3.058366195 |
| 202 | MBCR245 | 233.3883597 | 8.043881407 |
| 203 | MBCR246 | 120.5153707 | 2.534335489 |
| 204 | MBCR247 | 267.9333333 | 2.948085669 |
| 205 | MBCR248 | 358.0920635 | 1.472939937 |
| 206 | MBCR249 | 352.9571428 | 4.701624228 |
| 207 | MBCR250 | 272.7309524 | 1.745742764 |
| 208 | MBCR251 | 231.3354498 | 5.34310707 |
| 209 | MBCR252 | 281.0603174 | 7.338795132 |
| 210 | MBCR253 | 171.3407408 | 1.374344874 |
| 211 | MBCR254 | 168.2243387 | 2.507647604 |
| 212 | MBCR255 | 525.0824173 | 6.771458155 |
| 213 | MBCR256 | 327.8195767 | 5.003642242 |
| 214 | MBCR257 | 226.2370371 | 3.804239021 |
| 215 | MBCR258 | 97.58913313 | 0.882871129 |
| 216 | MBCR259 | 207.0180871 | 0.752237245 |
| 217 | MBCR260 | 173.3142857 | 1.322046257 |
| 218 | MBCR261 | 345.3936508 | 3.482209946 |
| 219 | MBCR262 | 306.2137566 | 1.613884504 |
| 220 | MBCR264 | 243.8539682 | 1.238594452 |
| 221 | MBCR265 | 588.3724868 | 0.909590154 |
| 222 | MBCR266 | 409.2310406 | 1.570016362 |
| 223 | MBCR267 | 231.3959436 | 0.327837754 |
| 224 | MBCR268 | 172.335097 | 0.576328413 |
| 225 | MBCR269 | 207.154321 | 0.419729906 |
| 226 | MBCR271 | 178.133157 | 0.303647329 |
| 227 | MBCR272 | 311.2283951 | 0.316286089 |
| 228 | MBCR273 | 357.4232804 | 0.402191051 |
| 229 | MBCR274 | 336.0841503 | 1.039925732 |
| 230 | MBCR275 | 335.3722223 | 0.692612529 |
| 231 | MBCR276 | 614.0497355 | 6.050417496 |
| 232 | MBCR277 | 274.1516755 | 0.320279708 |
| 233 | MBCR278 | 242.6569665 | 0.662982536 |
| 234 | MBCR279 | 265.324515 | 0.362303525 |
| 235 | MBCR280 | 165.6444444 | 0.518037256 |
| 236 | MBCR281 | 158.6685185 | 0.459479144 |
| 237 | MBCR282 | 144.162963 | 1.281211256 |
| 238 | MBCR283 | 185.0574074 | 0.575837514 |
| 239 | MBCR284 | 152.2826089 | 3.937571735 |
| 240 | MBCR285 | 139.3633157 | 0.270519979 |
| 241 | MBCR286 | 135.2364197 | 0.204756954 |
| 242 | MBCR287 | 348.8275132 | 0.969329052 |
| 243 | MBCR288 | 328.7055556 | 14.45736689 |
| 244 | MBCR289 | 400.2878307 | 0.893124084 |
| 245 | MBCR290 | 498.4092592 | 0.398176568 |
| 246 | MBCR291 | 355.1079366 | 0.2362422 |
| 247 | MBCR292 | 150.0561729 | 3.607685185 |
| 248 | MBCR293 | 295.3777777 | 2.826397663 |
| 249 | MBCR294 | 224.3858907 | 0.540410182 |
| 250 | MBCR295 | 58.01164021 | 0.324110652 |
| 251 | MBCR296 | 90.22883597 | 0.549403366 |
| 252 | MBCR297 | 191.0784832 | 0.439229513 |
| 253 | MBCR298 | 314.0458554 | 0.461013836 |
| 254 | MBCR299 | 97.3968254 | 0.487956653 |
| 255 | MBCR300 | 370.7786596 | 0.263620538 |
| 256 | MBCR301 | 342.6040564 | 0.439888448 |
| 257 | MBCR302 | 162.3716931 | 0.331316335 |
| 258 | MBCR303 | 199.835097 | 0.45935696 |
| 259 | MBCR304 | 209.9938272 | 0.902441199 |
| 260 | MBCR305 | 138.3007055 | 0.213828948 |
| 261 | MBCR306 | 219.6322751 | 0.789021731 |
| 262 | MBCR307 | 276.9777777 | 5.263764855 |
| 263 | MBCR308 | 162.4541446 | 0.489841214 |
| 264 | MBCR309 | 412.7420634 | 0.426251365 |
| 265 | MBCR310 | 270.6419753 | 0.5506535 |
| 266 | MBCR311 | 101.6838624 | 0.392625817 |
| 267 | MBCR312 | 240.6507937 | 1.063159944 |
| 268 | MBCR313 | 311.8558201 | 0.320905128 |
| 269 | MBCR314 | 405.8844797 | 0.53563665 |
| 270 | MBCR315 | 325.7257496 | 0.519731071 |
| 271 | MBCR316 | 164.8968254 | 0.410764925 |
| 272 | MBCR317 | 120.9771605 | 0.395904104 |
| 273 | MBCR318 | 353.6402116 | 0.798844821 |
| 274 | MBCR319 | 188.6490299 | 1.242289621 |
| 275 | MBCR320 | 161.2208995 | 0.318213467 |
| 276 | MBCR321 | 117.5820106 | 0.829181296 |
| 277 | MBCR322 | 124.8716931 | 0.360313963 |
| 278 | MBCR323 | 228.8650794 | 1.044137702 |
| 279 | MBCR324 | 103.5167548 | 0.53871664 |
| 280 | MBCR325 | 479.5573192 | 0.914523497 |
| 281 | MBCR326 | 351.9867725 | 0.909440343 |
| 282 | MBCR327 | 210.5537919 | 0.563846592 |
| 283 | MBCR328 | 301.5414462 | 0.528985286 |
| 284 | MBCR329 | 282.8553792 | 0.796585393 |
| 285 | MBCR330 | 649.7910053 | 0.473331717 |
| 286 | MBCR331 | 147.6878307 | 0.505466925 |
| 287 | MBCR332 | 343.9400353 | 0.554090834 |
| 288 | MBCR333 | 263.9179894 | 1.005707961 |
| 289 | MBCR334 | 335.3289242 | 0.795589 |
| 290 | MBCR335 | 391.1093474 | 0.473213445 |
| 291 | MBCR336 | 183.9973545 | 0.298087827 |
| 292 | MBCR337 | 285.3112875 | 0.435655145 |
| 293 | MBCR338 | 124.1472663 | 0.560535275 |
| 294 | MBCR339 | 95.29982363 | 1.569177198 |
| 295 | MBCR340 | 208.0198412 | 0.412669817 |
| 296 | MBCR341 | 102.5410053 | 0.201987793 |
| 297 | MBCR342 | 366.3694886 | 0.766417636 |
| 298 | MBCR343 | 190.3880071 | 0.964939444 |
| 299 | MBCR344 | 232.643739 | 0.433733386 |
| 300 | MBCR345 | 215.6728395 | 0.598700791 |
| 301 | MBCR346 | 494.2045856 | 0.470942628 |
| 302 | MBCR347 | 168.1604938 | 0.271857801 |
| 303 | NDS349 | 388.5548942 | 11.38280493 |
| 304 | NDS353 | 1618.75 | 8.213213395 |
| 305 | NDS354 | 596.9642858 | 2.645612699 |
| 306 | NDS356 | 124.8809524 | 6.799273232 |
| 307 | NDS358 | 154.0476191 | 17.36436725 |
| 308 | NDS359 | 152.2325983 | 2.745656448 |
| 309 | NDS360 | 385 | 19.34214785 |
| 310 | NDS361 | 121.0509259 | 8.300725092 |
| 311 | NDS363 | 1333.035714 | 14.00466747 |
| 312 | NDS364 | 298.0952381 | 14.55336524 |
| 313 | NDS366 | 236.7857143 | 33.79831381 |
| 314 | NDS367 | 546.3095238 | 21.49623147 |
| 315 | NDS368 | 330.2380953 | 23.49477693 |
| 316 | NDS369 | 1702.083333 | 14.85375107 |
| 317 | NDS370 | 677.8571429 | 23.56339981 |
| 318 | NDS371 | 610 | 11.00348322 |
| 319 | NDS372 | 138.5238095 | 11.23725502 |
| 320 | NDS374 | 984.8214286 | 5.088195922 |
| 321 | NDS375 | 795.8333333 | 34.3941943 |
| 322 | NDS376 | 311.0277778 | 52.5461952 |
| 323 | NDS377 | 783.9285714 | 8.980724347 |
| 324 | NDS378 | 828.5714286 | 41.65025324 |
| 325 | NDS379 | 1297.321429 | 28.15315317 |
| 326 | NDS380 | 743.75 | 8.605763931 |
| 327 | NDS381 | 595.297619 | 8.342127552 |
| 328 | NDS382 | 495.7142857 | 25.51431981 |
| 329 | NDS385 | 1380.654762 | 5.983710269 |
| 330 | NDS386 | 349.6428571 | 20.21976027 |
| 331 | NDS387 | 119.092381 | 2.327989604 |
| 332 | NDS389 | 449.8809524 | 1.96906192 |
| 333 | NDS395 | 369.5238095 | 17.68190965 |
| 334 | NDS396 | 267.7380952 | 46.60176431 |
| 335 | NDS397 | 340.3571429 | 12.42228909 |
| 336 | NDS398 | 164.7619048 | 7.060331174 |
| 337 | NDS400 | 458.2142857 | 11.94078489 |
| 338 | NDS401 | 221.2599207 | 6.667164089 |
| 339 | NDS402 | 87.93518518 | 91.83579821 |
| 340 | NDS403 | 342.1428571 | 17.26264931 |
| 341 | NDS404 | 223.0952381 | 24.8582022 |
| 342 | NDS405 | 370.1190476 | 5.820862706 |
| 343 | NDS406 | 138.4285714 | 1.851832491 |
| 344 | NDS408 | 452.8571428 | 161.7451071 |
| 345 | NDS410 | 28.43756614 | 13.63944425 |
| 346 | NDS411 | 468.9285714 | 11.55900536 |
| 347 | NDS412 | 452.8571429 | 32.39240918 |
| 348 | NDS413 | 794.3452381 | 36.90930945 |
| 349 | NDS414 | 229.1071428 | 5.341884499 |
| 350 | NDS415 | 445.7142857 | 10.61834596 |
| 351 | NDS416 | 898.5119048 | 6.789017669 |
| 352 | NDS417 | 897.0238095 | 28.27734367 |
| 353 | NDS418 | 1510.119048 | 37.36217594 |
| 354 | NDS419 | 197.2619048 | 3.037915552 |
| 355 | NDS420 | 845.297619 | 30.86084798 |
| 356 | NDS421 | 201.6666667 | 33.26704122 |
| 357 | NDS423 | 290.4728836 | 6.847611335 |
| 358 | NDS425 | 198.9485715 | 0 |
| 359 | NDS427 | 145.3028571 | 0.751012144 |
| 360 | NDS428 | 245.7980953 | 0.421444466 |
| 361 | NDS430 | 147.6118519 | 5.754288156 |
| 362 | NDS431 | 231.7866667 | 0.417035649 |
| 363 | NDS434 | 383.1087302 | 26.25333855 |
| 364 | NDS436 | 215.9722429 | 0 |
| 365 | NDS438 | 275.6580688 | 2.818361884 |
| 366 | NDS439 | 1115.773809 | 7.241980423 |
| 367 | NDS440 | 165.7666667 | 0 |
| 368 | NDS441 | 116.5155559 | 3.713879069 |
| 369 | NDS442 | 116.7069385 | 0 |
| 370 | NDS443 | 347.6571429 | 0 |
| 371 | NDS444 | 199.7790476 | 0 |
| 372 | NDS445 | 58.19973545 | 5.387203333 |
| 373 | NDS446 | 332.0580849 | 0 |
| 374 | NDS447 | 192.3885714 | 0.316744588 |
| 375 | NDS448 | 64.92952381 | 0 |
| 376 | NDS450 | 76.89142857 | 0.339343977 |
| 377 | NDS451 | 805.8333333 | 34.31586437 |
| 378 | NDS452 | 146.4252645 | 27.79664934 |
| 379 | NDS453 | 99.25714286 | 0 |
| 380 | NDS454 | 64.77714286 | 30.743624 |
| 381 | NDS455 | 63.86285715 | 3.766744902 |
| 382 | NDS456 | 77.95809524 | 0.254193899 |
| 383 | NDS457 | 107.0552381 | 0.298448293 |
| 384 | NDS458 | 57.08862434 | 53.23616126 |
| 385 | NDS459 | 154.8266666 | 0 |
| 386 | NDS460 | 205.8125748 | 0 |
| 387 | NDS461 | 59.59619047 | 0 |
| 388 | NDS462 | 176.9219047 | 0.348577827 |
| 389 | NDS463 | 41.00571429 | 0.134189882 |
| 390 | NDS464 | 199.0933333 | 0.297071121 |
| 391 | NDS465 | 34.45740741 | 1.468147276 |
| 392 | NDS466 | 45.57714285 | 0.224457072 |
| 393 | NDS467 | 276.4351684 | 0.243876676 |
| 394 | NDS468 | 193.925948 | 0.352274711 |
| 395 | NDS469 | 40.33333333 | 141.9555167 |
| 396 | NDS470 | 74.83428572 | 0.223784428 |
| 397 | NDS471 | 167.7086736 | 0.395532871 |
| 398 | NDS472 | 72 | 8.18705871 |
| 399 | NDS473 | 185.4552381 | 0.900661211 |
| 400 | NDS476 | 70.56761905 | 0.267350499 |
| 401 | NDS477 | 111.0933333 | 0.348069375 |
| 402 | NDS478 | 0 | 0 |
| 403 | NDS480 | 83.28600192 | 0.244689108 |
| 404 | NDS481 | 63.51719577 | 3.215942368 |
| 405 | NDS482 | 85.88190477 | 0.36540728 |
| 406 | NDS483 | 68.66285714 | 0.290825333 |
| 407 | NDS484 | 63.10095238 | 0 |
| 408 | NDS485 | 163.5961905 | 0.764838047 |
| 409 | NDS486 | 207.0933333 | 0.342312971 |
| 410 | NDS487 | 295.2838096 | 1.087967954 |
| 411 | NDS488 | 96.10714286 | 1.463802426 |
| 412 | NDS489 | 252.807619 | 0.237116494 |
| 413 | NDS490 | 150.1104762 | 0 |
| 414 | NDS491 | 49.99619047 | 0.284129345 |
| 415 | NDS492 | 66.68190476 | 0 |
| 416 | NDS493 | 235.9504337 | 0 |
| 417 | NDS494 | 186.1904762 | 13.83125774 |
| 418 | NDS495 | 39.70285714 | 0.222340658 |
| 419 | NDS496 | 106.1600529 | 4.24375191 |
| 420 | NDS497 | 350.0825721 | 1.251895832 |
| 421 | NDS498 | 320.007619 | 0 |
| 422 | NDS499 | 65.92 | 5.571825321 |
| 423 | NDS500 | 216.7695239 | 0 |
| 424 | NDS501 | 975.4761905 | 209.1485709 |
| 425 | NDS503 | 478.452381 | 17.52852122 |
| 426 | NDS504 | 597.5 | 26.2477295 |
| 427 | NDS505 | 298.0952381 | 63.16273936 |
| 428 | NDS506 | 43.08068783 | 8.584555755 |
| 429 | NDS507 | 407.0238095 | 87.31502203 |
| 430 | NDS508 | 47.9484127 | 2.802589272 |
| 431 | NDS509 | 231.8185185 | 2.918409879 |
| 432 | NDS510 | 98.45833334 | 1.830911742 |
| 433 | NDS511 | 254.5801414 | 0.693677963 |
| 434 | NDS512 | 101.1296296 | 5.692164331 |
| 435 | NDS513 | 95.17857143 | 0.974700398 |
| 436 | NDS514 | 980.3648148 | 16.40464395 |
| 437 | NDS515 | 185.8185185 | 16.72500477 |
| 438 | NDS516 | 461.9666667 | 3.021767598 |
| 439 | NDS517 | 164.7444444 | 18.3245581 |
| 440 | NDS518 | 426.2351852 | 3.983544919 |
| 441 | NDS519 | 186.7074074 | 15.62123303 |
| 442 | NDS520 | 293.7074074 | 7.181504524 |
| 443 | NDS521 | 417.2351852 | 36.82290588 |
| 444 | NDS523 | 373.0777778 | 4.063806276 |
| 445 | NDS524 | 91.28240741 | 2.645526274 |
| 446 | NDS525 | 220.9666667 | 2.197661443 |
| 447 | NDS526 | 147.5851852 | 7.319368341 |
| 448 | NDS527 | 337.7537037 | 3.1468173 |
| 449 | NDS528 | 376.3525132 | 2.312822403 |
| 450 | NDS529 | 792.9388889 | 11.31063802 |
| 451 | NDS530 | 714.587037 | 6.0797452 |
| 452 | NDS531 | 1387.087037 | 2.246048541 |
| 453 | NDS532 | 1126.753704 | 14.94515678 |
| 454 | NDS533 | 945.7722222 | 15.19337259 |
| 455 | NDS534 | 917.7259259 | 3.651388309 |
| 456 | NDS535 | 346.0777778 | 3.868802963 |
| 457 | NDS536 | 354.3462963 | 24.24826923 |
| 458 | NDS537 | 258.1518519 | 3.863245555 |
| 459 | NDS539 | 151.8185185 | 13.42913079 |
| 460 | NDS540 | 231.8925926 | 18.30671674 |
| 461 | NDS541 | 614.8809524 | 0 |
| 462 | NDS542 | 40.25410164 | 0 |
| 463 | NDS543 | 143.3740741 | 2.532411241 |
| 464 | NDS544 | 225.5222222 | 2.216673151 |
| 465 | NDS545 | 93.47037037 | 58.72471777 |
| 466 | NDS546 | 152.3740741 | 4.853344892 |
| 467 | NDS547 | 151.0714286 | 0 |
| 468 | NDS548 | 226.9666667 | 1.593556342 |
| 469 | NDS550 | 177.9296296 | 8.533852727 |
| 470 | NDS551 | 146.9666667 | 8.9516083 |
| 471 | NDS552 | 209.9296296 | 19.71986566 |
| 472 | NDS553 | 861.1904762 | 0.55986775 |
| 473 | NDS554 | 299.8809524 | 0 |
| 474 | NDS555 | 348.8555556 | 2.908932729 |
| 475 | NDS556 | 357.2722222 | 2.643300054 |
| 476 | NDS557 | 363.0777778 | 31.13156514 |
| 477 | NDS558 | 599.8809523 | 0 |
| 478 | NDS559 | 784.4047619 | 0 |
| 479 | NDS560 | 1050.47619 | 0 |
| 480 | NDS561 | 805.8333333 | 0.660067861 |
| 481 | NDS562 | 365.4761905 | 0 |
| 482 | NDS563 | 1412.919974 | 0 |
| 483 | NDS564 | 161.9404762 | 3.440530061 |
| 484 | NDS565 | 712.5 | 0 |
| 485 | NDS566 | 591.6666667 | 0.788484195 |
| 486 | NDS567 | 702.3809524 | 0 |
| 487 | NDS568 | 221.2202381 | 2.187977768 |
| 488 | NDS569 | 0 | 12.23034858 |
| 489 | NDS570 | 1931.25 | 0 |
| 490 | NDS572 | 791.3690476 | 9.918754513 |
| 491 | NDS573 | 169.4801588 | 3.182592545 |
| 492 | NDS574 | 1107.142859 | 13.38921068 |
| 493 | NDS575 | 303.2738095 | 8.262692778 |
| 494 | NDS576 | 330.6325476 | 69.93123952 |
| 495 | NDS578 | 1990.77381 | 0 |
| 496 | NDS581 | 87.38095238 | 2.383622169 |
| 497 | NDS582 | 749.702381 | 0 |
| 498 | NDS583 | 513.3928572 | 0 |
| 499 | NDS586 | 885.1190476 | 0 |
| 500 | NDS587 | 663.6904762 | 0 |
| 501 | NDS589 | 783.9285714 | 0 |
| 502 | NDS590 | 619.0476191 | 0 |
| 503 | NDS591 | 599.702381 | 0 |
| 504 | NDS594 | 764.8809526 | 0 |
| 505 | NDS595 | 386.8154758 | 0 |
| 506 | NDS597 | 1072.619047 | 0 |
| 507 | NDS598 | 30.10555555 | 15.33226413 |
| 508 | NDS599 | 1356.845238 | 0 |
| 509 | NDS600 | 571.5151515 | 46.20298093 |
| 510 | NDS602 | 104.7407407 | 3.066907574 |
| 511 | NDS607 | 436.2528237 | 41.70222326 |
| 512 | NDS614 | 129.2949735 | 2.470343069 |
| 513 | NDS615 | 67.19444444 | 50.47265786 |
| 514 | NDS618 | 181.2045628 | 37.05669668 |
| 515 | NDS619 | 399.084357 | 59.1502385 |
| 516 | NDS620 | 160.2314815 | 2.031191943 |
| 517 | NDS622 | 420.5369139 | 40.13940654 |
| 518 | NDS623 | 345.3659799 | 53.51514623 |
| 519 | NDS625 | 468.4049178 | 47.25880569 |
| 520 | NDS626 | 110.2962963 | 2.701466773 |
| 521 | NDS628 | 277.9062363 | 31.66339431 |
| 522 | NDS629 | 99.15277778 | 8.224631894 |
| 523 | NDS630 | 549.4243077 | 47.6060017 |
| 524 | NDS631 | 225.7286355 | 37.21675498 |
| 525 | NDS633 | 239.6743401 | 6.434601592 |
| 526 | NDS634 | 188.2572751 | 59.70752476 |
| 527 | NDS636 | 189.8581645 | 19.08511568 |
| 528 | NDS637 | 440.739459 | 37.9675087 |
| 529 | NDS638 | 478.4304419 | 48.75669581 |
| 530 | NDS639 | 483.1743627 | 28.22621431 |
| 531 | NDS640 | 67.32842959 | 24.55397569 |
| 532 | NDS641 | 747.0246824 | 37.50820098 |
| 533 | NDS642 | 146.5575397 | 4.678164825 |
| 534 | NDS643 | 198.1342593 | 2.491174481 |
| 535 | NDS645 | 115.6666667 | 75.21237063 |
| 536 | NDS646 | 824.3079804 | 35.61720055 |
| 537 | NDS668 | 1811.800239 | 193.4671665 |
| 538 | NDS669 | 1089.323229 | 54.67314733 |
| 539 | NDS670 | 193.8153901 | 5.027830252 |
| 540 | NDS671 | 283.0441373 | 42.52779207 |
| 541 | NDS672 | 411.923669 | 47.5842115 |
| 542 | NDS674 | 272.8770503 | 38.3866827 |

**Supplementary Table 6. Optimization runs of an extraction method using research surface methodology – box behnken design with TPC, TFC, TAC, DPPH scavenging activity, and FRAP as responses.**

| **Run** | **Factors** | | | **Responses** | | | | |
| --- | --- | --- | --- | --- | --- | --- | --- | --- |
|  | **Ethanol, %** | **Extraction temperature, °C** | **Extraction time, min** | **TPC, mg GAE/100g DM** | **TFC, mg CE/100g DM** | **TAC, mgC3G/100g DM** | **DPPH, mg TE/100g DM** | **FRAP, mg TE/100g DM** |
| 1 | 75 | 45 | 52.5 | 1826.3 | 1385.02 | 3530.67 | 0.797103 | 2.6339 |
| 2 | 100 | 30 | 52.5 | 136.076 | 30.00 | 384.232 | 0.244921 | 0.118082 |
| 3 | 50 | 45 | 45 | 2218.89 | 1720.46 | 2776.79 | 0.634312 | 2.68886 |
| 4 | 75 | 45 | 52.5 | 1770.74 | 1110.76 | 3599.04 | 0.845006 | 2.75743 |
| 5 | 75 | 60 | 60 | 2000.37 | 1568.57 | 3205.07 | 0.743723 | 2.62447 |
| 6 | 50 | 60 | 52.5 | 2681.85 | 2273.21 | 2499.81 | 0.586098 | 2.73067 |
| 7 | 75 | 30 | 60 | 1744.81 | 893.46 | 3497.98 | 0.804987 | 2.62635 |
| 8 | 75 | 60 | 45 | 1696.67 | 931.435 | 3388.26 | 0.840145 | 2.41685 |
| 9 | 75 | 45 | 52.5 | 2267.04 | 1013.71 | 3143.39 | 0.699666 | 2.96689 |
| 10 | 50 | 30 | 52.5 | 2085.56 | 1496.84 | 2645.2 | 0.59558 | 2.28087 |
| 11 | 75 | 45 | 52.5 | 2048.52 | 1028.48 | 3200.41 | 0.72147 | 2.58961 |
| 12 | 100 | 45 | 45 | 851.266 | 118.889 | 576.48 | 0.280213 | 0.111306 |
| 13 | 75 | 30 | 45 | 1992.96 | 1385.02 | 3412.84 | 0.756669 | 2.6918 |
| 14 | 75 | 45 | 52.5 | 990.506 | 118.889 | 3344.09 | 0.773834 | 2.51669 |
| 15 | 100 | 60 | 52.5 | 1328.06 | 944.815 | 1122.88 | 0.23169 | 0.670739 |
| 16 | 100 | 45 | 60 | 1463.08 | 241.111 | 714.486 | 0.23237 | 0.227553 |
| 17 | 50 | 45 | 60 | 2126.3 | 1330.17 | 2686.9 | 0.590216 | 2.45961 |

**Supplementary Table 7. Developed response surface equations for predicting the TPC, TFC, TAC, DPPH scavenging activity, and FRAP.**

| **Responses** | **R^2^** | **Predicted R^2^** | **Adjusted R^2^** | **Final Equation in terms of Coded Factors** | **Final Equation in terms of Actual Factors** |
| --- | --- | --- | --- | --- | --- |
| TPC | 0.6563 | 0.4323 | 0.5770 | 1719.35 - 666.76*A + 218.44*B + 71.85*C | 2561.39082 - 26.67059*Ethanol + 14.56287* Extraction Temperature + 9.57957*Extraction Time |
| TFC | 0.6811 | 0.4831 | 0.6075 | 1034.75 - 685.73*A + 239.09*B - 15.31*C | 2481.86974 - 27.42933*Ethanol + 15.93925*Extraction Temperature - 2.04155*Extraction TIme |
| TAC | 0.9852 | 0.8758 | 0.9661 | 3363.52 - 976.33*A + 34.37*B - 6.24*C + 221.01*AB + 56.97*AC - 67.08*BC - 1693.93A^2^ - 6.56B^2^ + 19.07C^2^ | -6360.11650 + 325.01659*Ethanol - 7.97547*Extraction Temperature - 32.39643*Extraction Time + 0.58936*Ethanol*Extraction Temperature + 0.30386*Ethanol*Extraction Time - 0.59629*Extraction Temperature*Extraction Time - 2.71029*Ethanol^2^ - 0.029147*Extraction Temperature^2^ + 0.33912*Extraction TIme^2^ |
| DPPH | 0.9823 | 0.9633 | 0.9595 | 0.7674 - 0.1771*A + 0.0001*B - 0.0175*C + 0.0009*AB + 0.0009*AC - 0.0362*BC - 0.3525 A^2^ - 0.0004B^2^ + 0.0193C^2^ | -1.59463 + 0.077883*Ethanol + 0.017218*Extraction Temperature - 0.023578*Extraction Time - 2.49933E-006*Ethanol*Extraction Temperature - 4.99600E-006*Ethanol*Extraction Time - 3.21644E-004*Extraction Temperature*Extraction Time - 5.63957E-004*Ethanol^2^ - 1.64511E-006*Extraction Temperature^2^ + 3.43740E-004*Extraction Time^2^ |
| FRAP | 0.9779 | 0.7058 | 0.9495 | 2.69 - 1.13*A + 0.0907*B + 0.0036*C + 0.0257*AB- + 0.0864*AC + 0.0683*BC - 1.23A^2^ - 0.0124 B^2^ - 0.0906 C^2^ | -6.36574 + 0.22287*Ethanol - 0.025998*Extraction Temperature + 0.10784*Extraction Time + 6.85713E-005*Ethanol*Extraction Temperature + 4.60663E-004*Ethanol*Extraction Time + 6.06822E-004*Extraction Temperature*Extraction TIme - 1.96868E-003*Ethanol^2^ - 5.50633E-005*Extraction Temperature^2^ - 1.61151E-003*Extraction Time^2^ |

**Supplementary Table 8. ANOVA for the Research Surface Linear and Polynomial Equations.**

| **Response variables** | **Source** | **df** | **Sum of Squares** | **Mean Square** | **F-value** | **p-value** |
| --- | --- | --- | --- | --- | --- | --- |
| TPC | Model | 3 | 3.980E+006 | 1.327E+006 | 8.28 | 0.0025^s^ |
|  | Lack of fit | 9 | 1.149E+006 | 1.277E+005 | 0.55 | 0.7928^ns^ |
|  | Pure error | 4 | 9.348+005 | 2.337E+005 |  |  |
|  | Residual | 13 | 2.084E+006 | 1.603E2+005 |  |  |
| TFC | Model | 3 | 4.221E+006 | 1.407E+006 | 9.26 | 0.0015^s^ |
|  | Lack of fit | 9 | 1.062E+006 | 1.180E+005 | 0.52 | 0.8117^ns^ |
|  | Pure error | 4 | 9.143E+005 | 2.286E+005 |  |  |
|  | Residual | 13 | 1.976E+006 | 1.520E+005 |  |  |
| TAC | Model | 9 | 2.001E+007 | 2.223E+006 | 51.70 | <0.0001^s^ |
|  | Lack of fit | 3 | 1.421E+005 | 47369.39 | 1.19 | 0.4186^ns^ |
|  | Pure error | 4 | 1.588E+005 | 39712.10 |  |  |
|  | Residual | 7 | 3.010E+005 | 42993.80 |  |  |
| DPPH | Model | 9 | 0.78 | 0.087 | 43.08 | <0.0001^s^ |
|  | Lack of fit | 3 | 4.984E-004 | 1.661E-004 | 0.049 | 0.9838^ns^ |
|  | Pure error | 4 | 0.014 | 3.411E-003 |  |  |
|  | Residual | 7 | 0.014 | 2.020E-003 |  |  |
| FRAP | Model | 9 | 16.82 | 1.87 | 34.41 | <0.0001^n^ |
|  | Lack of fit | 3 | 0.26 | 0.085 | 2.74 | 0.1774^ns^ |
|  | Pure error | 4 | 0.12 | 0.031 |  |  |
|  | Residual | 7 | 0.38 | 0.054 |  |  |

s= significant; ns= not significant

**Supplementary Table 9. Results of the confirmatory runs that were conducted using optimized conditions were obtained from the research surface method (51.244% ethanol, 60^o^C, and 1 hour extraction time).**

| **Analysis** | **Predicted Mean** | **Predicted Median** | **Observed** | **Std Dev** | **n** | **SE Pred** | **95% PI low** | **Data Mean** | **95% PI high** |
| --- | --- | --- | --- | --- | --- | --- | --- | --- | --- |
| TPC, mg GAE/100g DM | 2643.33 | 2643.33 | 3376.666 | 400.36 | 5 | 315.684 | 1961.34 | 3376.67 | 3325.33 |
| TFC, mg CE/100g DM | 1910.25 | 1910.25 | 2006.294 | 389.879 | 5 | 307.419 | 1246.11 | 2006.29 | 2574.39 |
| TAC, mg C3G/100g DM | 2470.83 | 2470.83 | 2608.47 | 207.35 | 5 | 252.464 | 1873.85 | 2608.47 | 3067.81 |
| DPPH, mg TE/100g DM | 0.584375 | 0.584375 | 0.5894122 | 0.0449479 | 5 | 0.0547275 | 0.454965 | 0.589412 | 0.713785 |
| FRAP, mg TE/100g DM | 2.6076 | 2.6076 | 3.128102 | 0.233054 | 5 | 0.283761 | 1.93661 | 3.1281 | 3.27859 |

**Supplementary Table 10. Phenolic compounds, antioxidant components, and capacities of the Kintuman, Balatinao, and Ketan Hitam RBEs and MRBEs.**

|  | **Kintuman rice bran extract** | **Balatinao rice bran extract** | **Ketan Hitam rice bran extract** | **Kintuman spray-dried rice bran extract** | **Balitanao spray-dried rice bran extract** | **Ketan Hitam spray-dried rice bran extract** |  |
| --- | --- | --- | --- | --- | --- | --- | --- |
| **Antioxidant components and capacities** | | | | | | | |
| TPC, mg/100g | 1499.96 ± 7.14 aA | 1538.24 ± 213.46 aA | 894.31 ± 4.59 bA | 191.04 ± 2.11 bB | 202.53 ± 1.43 bB | 230.41 ± 10.99 aB |  |
| TFC, mg/ 100g | 817.46 ± 85.61 aA | 519.14 ± 0.82 bA | 277.78 ± 56.61 cA | 182.60 ± 1.34 aB | 176.69 ± 4.17 aB | 192.69 ± 16.49 aA |  |
| TPAC, mg/100g | 214.59 ± 9.45 aA | 207.81 ± 7.48 aA | 210.56 ± 8.05 aA | 11.48 ± 0.03 bB | 14.97 ± 1.51 aB | 15.24 ± 0.10 aB |  |
| TAC, mg Cyn-3-Glucoside/ 100 g | 184.73 ± 10.42 bA | 46.56 ± 1.95 cA | 629.66 ± 24.97 aA | 48.19 ± 6.84 cB | 151.35 ± 6.85 bA | 216.36 ± 7.22 aB |  |
| DPPH, mg TE/g | 665.46 ± 7.33 aA | 229.16 ± 23.74 cB | 453.24 ± 26.97 bB | 490.76 ± 4.33 bB | 402.15 ± 3.09 cA | 508.57 ± 2.42 aA |  |
| ABTS, mg TE/g | 823.66 ± 9.51 bA | 1349.82 ± 1.36 aA | 558.18 ± 33.48 cA | 278.48 ± 46.34 cB | 441.83 ± 8.27 aB | 335.14 ± 6.77 bB |  |
| FRAP, mg TE/g | 94.10 ± 4.40 aA | 93.16 ± 3.71 aA | 98.84 ± 5.27 aA | 86.19 ± 3.20 aA | 79.62 ± 2.84 bB | 88.95 ± 1.21 aB |  |
| **Phenolic compounds** | | | | | | | |
| Apigenin, mg/100g | 0.041 ± 0.001 aA | 0.049 ± 0.005 aA | 0.040 ± 0.005 aA | 0.000 ± 0.000 B | 0.000 ± 0.000 B | 0.000 ± 0.000 B |  |
| Caffeic Acid, mg/100g | 0.381 ± 0.165 aA | 0.170 ± 0.061 abA | 0.081 ± 0.016 bA | 0.185 ± 0.054 aA | 0.000 ± 0.000 bB | 0.123 ± 0.038 aA |  |
| Catechin hydrate, mg/100g | 0.000 ± 0.000 B | 0.000 ± 0.000 | 0.000 ± 0.000 | 1.080 ± 0.071 aA | 0.000 ± 0.000 b | 0.000 ± 0.000 b |  |
| Chlorogenic Acid, mg/100g | 0.063 ± 0.002 aB | 0.041 ± 0.035 aA | 0.063 ± 0.004 aA | 0.091 ± 0.011 aA | 0.070 ± 0.008 aA | 0.048 ± 0.043 aA |  |
| Ellagic Acid, mg/100g | 0.200 ± 0.036 cA | 2.967 ± 0.310 aA | 1.073 ± 0.080 bA | 0.000 ± 0.000 bB | 0.000 ± 0.000 bB | 0.180 ± 0.047 aB |  |
| Epicatechin, mg/100g | 1.512 ± 0.086 aA | 1.338 ± 0.086 aA | 0.842 ± 0.060 bA | 0.476 ± 0.048 aB | 0.318 ± 0.035 bB | 0.494 ± 0.045 aB |  |
| Gallic Acid, mg/100g | 0.067 ± 0.036 bA | 0.262 ± 0.002 aA | 0.146 ± 0.060 bA | 0.047 ± 0.016 aA | 0.253 ± 0.134 aA | 0.206 ± 0.120 aA |  |
| Isovitexin, mg/100g | 0.051 ± 0.004 bA | 0.164 ± 0.037 aA | 0.192 ± 0.021 aA | 0.000 ± 0.000 bB | 0.055 ± 0.018 aB | 0.068 ± 0.012 aB |  |
| Luteolin 7-Glucoside, mg/100g | 0.089 ± 0.011 bA | 0.306 ± 0.068 aA | 0.234 ± 0.037 aA | 0.000 ± 0.000 bB | 0.065 ± 0.016 aB | 0.000 ± 0.000 bB |  |
| Naringenin, mg/100g | 0.088 ± 0.006 cA | 0.250 ± 0.005 aA | 0.157 ± 0.009 bA | 0.067 ± 0.023 aA | 0.075 ± 0.005 aB | 0.096 ± 0.005 aB |  |
| p-Coumaric Acid, mg/100g | 0.810 ± 0.068 aA | 0.725 ± 0.069 aA | 0.420 ± 0.017 bA | 0.468 ± 0.055 aB | 0.263 ± 0.029 cB | 0.359 ± 0.022 bB |  |
| Petunidin 3-Glucoide chloride, mg/100g | 0.000 ± 0.000 c | 0.069 ± 0.008 bA | 0.099 ± 0.017 aB | 0.000 ± 0.000 b | 0.081 ± 0.052 abA | 0.157 ± 0.023 aA |  |
| Quercetin, mg/100g | 0.103 ± 0.013 cA | 6.243 ± 0.370 aA | 3.603 ± 0.670 bA | 0.078 ± 0.008 aA | 0.117 ± 0.048 aB | 0.142 ± 0.005 aB |  |
| Rutin Hydrate, mg/100g | 0.000 ± 0.000 b | 0.000 ± 0.000 b | 0.152 ± 0.053 aA | 0.000 ± 0.000 | 0.000 ± 0.000 | 0.000 ± 0.000 B |  |
| Sinapic Acid, mg/100g | 0.651 ± 0.081 bA | 0.907 ± 0.043 aA | 0.588 ± 0.140 bA | 0.231 ± 0.001 bB | 0.187 ± 0.015 bB | 0.524 ± 0.039 aA |  |
| Syringic Acid, mg/100g | 0.051 ± 0.009 aA | 0.047 ± 0.025 aA | 0.022 ± 0.007 aA | 0.021 ± 0.001 abB | 0.012 ± 0.012 bA | 0.049 ± 0.017 aA |  |
| Trans-Ferulic Acid, mg/100g | 1.642 ± 0.121 aA | 1.064 ± 0.065 bA | 0.599 ± 0.068 cA | 0.565 ± 0.008 aB | 0.216 ± 0.024 bB | 0.532 ± 0.042 aA |  |
| Vanillin, mg/100g | 0.237 ± 0.096 aA | 0.202 ± 0.048 aB | 0.247 ± 0.054 aB | 0.170 ± 0.018 bA | 0.504 ± 0.080 aA | 0.513 ± 0.077 aA |  |
| Vitexin, mg/100g | 0.036 ± 0.004 bA | 0.153 ± 0.045 aA | 0.179 ± 0.023 aA | 0.027 ± 0.006 aA | 0.038 ± 0.016 aB | 0.051 ± 0.014 aB |  |

Values are mean ± standard deviation of three independent determinations (n = 3); Small letters denote a significant difference (p < 0.05) in rice bran, and spray-dried rice bran extract, while capital letters denote significant differences (p <0.05) within genotypes.

**Supplementary Table 11. Nephrotoxicity evaluation of the pigmented rice bran extracts**

| Samples | Cell_lines | Mean (1/IC50) | SD |
| --- | --- | --- | --- |
| Encapsulated_black_bran (Ketan Hitam) | Colon_cancer | 0.00276984 | 0.00010712 |
| Encapsulated_red_bran (Kintuman) | Colon_cancer | 0.00296172 | 0.00038553 |
| Non-Encapsulated_black_ bran (Ketan Hitam) | Colon_cancer | 0.00045678 | 5.35E-05 |
| Non-Encapsulated_red_bran (Kintuman) | Colon_cancer | 0.00071471 | 9.12E-05 |
| Encapsulated_black_bran (Ketan Hitam) | Lung_cancer | 0.00316721 | 0.00031141 |
| Encapsulated_red_bran (Kintuman) | Lung_cancer | 0.00312473 | 3.85E-05 |
| Non-Encapsulated_black_bran (Ketan Hitam) | Lung_cancer | 0.00177923 | 0.00011196 |
| Non-Encapsulated_red_bran (Kintuman) | Lung_cancer | 0.0018425 | 0.00052106 |

**Supplementary Table 12. Nephrotoxicity evaluation of the pigmented rice bran extracts**

| Sample | Concentration (ppm) | Cytotoxicity (%) | | | Cytotoxicity Interpretation |
| --- | --- | --- | --- | --- | --- |
|  |  | Trial 1 | Trial 2 | Trial 3 |  |
| Non-encapsulated Ketan Hitam black rice bran extract | 44 ppm | 0.08 | <0 | <0 | Safe |
| Microencapsulated Ketan Hitam black rice bran extract | 200 | 2.02 | 0.62 | <0 | Safe |
| Non-encapsulated Kintuman red rice bran extract | 315 | 0.41 | 3.05 | <0 | Safe |
| Microencapsulated Kintuman red rice bran extract | 345 | <0% | 0.52 | <0 | Safe |
